# Pathological tau alters head direction signaling and induces spatial disorientation

**DOI:** 10.1016/j.celrep.2025.116610

**Published:** 2025-11-22

**Authors:** Shan Jiang, Sara Hijazi, Barbara Sárkány, Verena G. Gautsch, Patrick A. LaChance, Michael E. Hasselmo, David Bannerman, Tim J. Viney

**Affiliations:** 1 Department of Pharmacology, University of Oxford, Oxford, UK; 2 Center for Systems Neuroscience, Boston University, Boston, MA, USA; 3 Department of Experimental Psychology, University of Oxford, Oxford, UK; 4 Present address: Cancer Research UK Cambridge Institute, University of Cambridge, Cambridge, UK; 5 These authors contributed equally; 6 Lead contact

## Abstract

Spatial disorientation is emerging as an early cognitive biomarker of dementia, but the underlying neural mechanisms remain undefined. The anterodorsal nucleus of the thalamus (ADn) exhibits early and selective vulnerability to pathological misfolded forms of tau, a major hallmark of Alzheimer’s disease. As the ADn contains a high density of head direction (HD) cells, we hypothesized that ptau disrupts HD cell activity, promoting spatial disorientation. To test this, we virally expressed human mutant tau in the ADn of adult wild-type and C1ql2-Cre mice. ADn-tau mice were defined by phosphorylated and oligomeric forms of tau in ADn somata and axon terminals. During initial spatial learning, ADn-tau mice exhibited increased looping behavior, indicative of spatial disorientation. Using *in vivo* extracellular recordings, we found that ADn cells had reduced directionality, lower directional coherence, and altered burst firing. These findings suggest that early alterations in HD signaling are predictive of future cognitive decline.

## In brief

Jiang and Hijazi et al. show that an early cognitive biomarker of dementia, spatial disorientation, can be induced in mice by expressing pathological tau in the anterodorsal nucleus of the thalamus. This alters the activity of head direction cells in this nucleus, affecting the animal’s sense of direction during spatial learning.

## Graphical Abstract

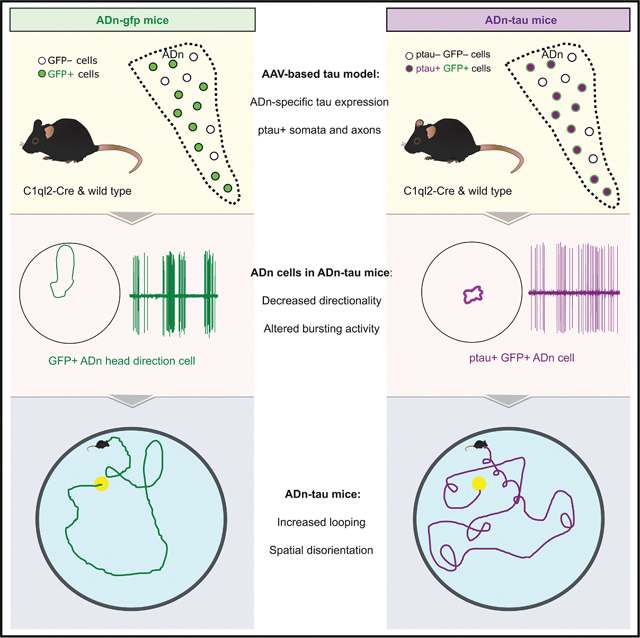

## INTRODUCTION

Spatial disorientation can be defined as a transient lack of awareness of one’s head direction with respect to spatial cues. Bouts of disorientation may occur prior to mild memory impairments.^1–7^ Therefore, disorientation has been suggested to be an early cognitive biomarker of dementia.^2,8^ However, the cause of these early behavioral changes remains unclear. Neurodegeneration and dementia are associated with the accumulation of pathological forms of tau (ptau). The Braak tau staging system, comprising stages I–VI, has been widely adopted to classify the progression of ptau in relation to Alzheimer’s disease, the most common form of dementia.^9^ The distribution of ptau in the cerebral cortex correlates with cognitive impairment, including memory loss from Braak stage III.^10–12^ We recently found that the human anterodorsal thalamic nucleus (ADn) accumulates ptau as early as “pre-Braak” stage 0,^13^ when cognitive impairments are not expected. The ADn was also found to exhibit extensive neurodegeneration in Alzheimer’s disease.^14^ In stark contrast, the adjacent anteroventral (AV) and mediodorsal thalamic nuclei remain unaffected at early Braak stages.^13,15^

The ADn contains a high density of head direction (HD) cells.^16^ HD cells are defined by an abrupt increase in firing when the head is oriented in a particular direction; i.e., their preferred firing direction.^17^ They are distributed across interconnected brain regions, including the lateral mammillary nucleus, anterior thalamic nuclei, dorsomedial striatum (DMS), retrosplenial cortex, postsubiculum (PoS), and entorhinal cortex.^16–24^ This HD network is essential for spatial orientation and navigation alongside grid cells, border cells, place cells, and other kinds of spatially modulated cells, forming a cognitive map of space.^25–28^ As a major node within this HD network, the ADn projects extensively to other brain areas that are also enriched in HD cells, including the granular retrosplenial cortex (RSg) and PoS.^20,29–34^

The ADn is traditionally grouped with the AV and anteromedial thalamic nuclei, forming the anterior thalamic nuclear group (ATN). Due to this grouping, lesion studies have largely overlooked the specific contributions of distinct nuclei. It is well established that ATN lesions in rodents cause spatial memory deficits.^35–39^ Only recently, with the development of more specific targeting techniques, has selective inhibition of the ADn been shown to impair spatial working memory in mice.^40–42^

Path integration enables individuals to maintain their position and orientation in space by continuously updating their sense of location based on self-motion cues (such as vestibular, proprioceptive, and motor information). Impairments in path integration can be detected in individuals with preclinical Alzheimer’s disease and mild cognitive impairment (MCI).^43,44^ Path integration errors are a result of poor angular estimation rather than distance estimation,^7^ which correlates with levels of ptau in the medial temporal lobe.^44^ This raises the question of whether selective accumulation of ptau in the human ADn at early stages could be detrimental to the functioning of HD cells, providing a neural basis for impaired path integration.^45,46^

To model early stages of accumulation of ptau within the HD network, we virally expressed mutant human tau in the ADn of both wild-type and C1ql2-Cre mice (ADn-tau mice). Our behavioral and physiological analysis of these mice facilitates a cross-validation of an early cognitive biomarker for dementia, promoting earlier detection and improved intervention strategies.

## RESULTS

### Mapping ptau in the HD network

**Figure 1.**
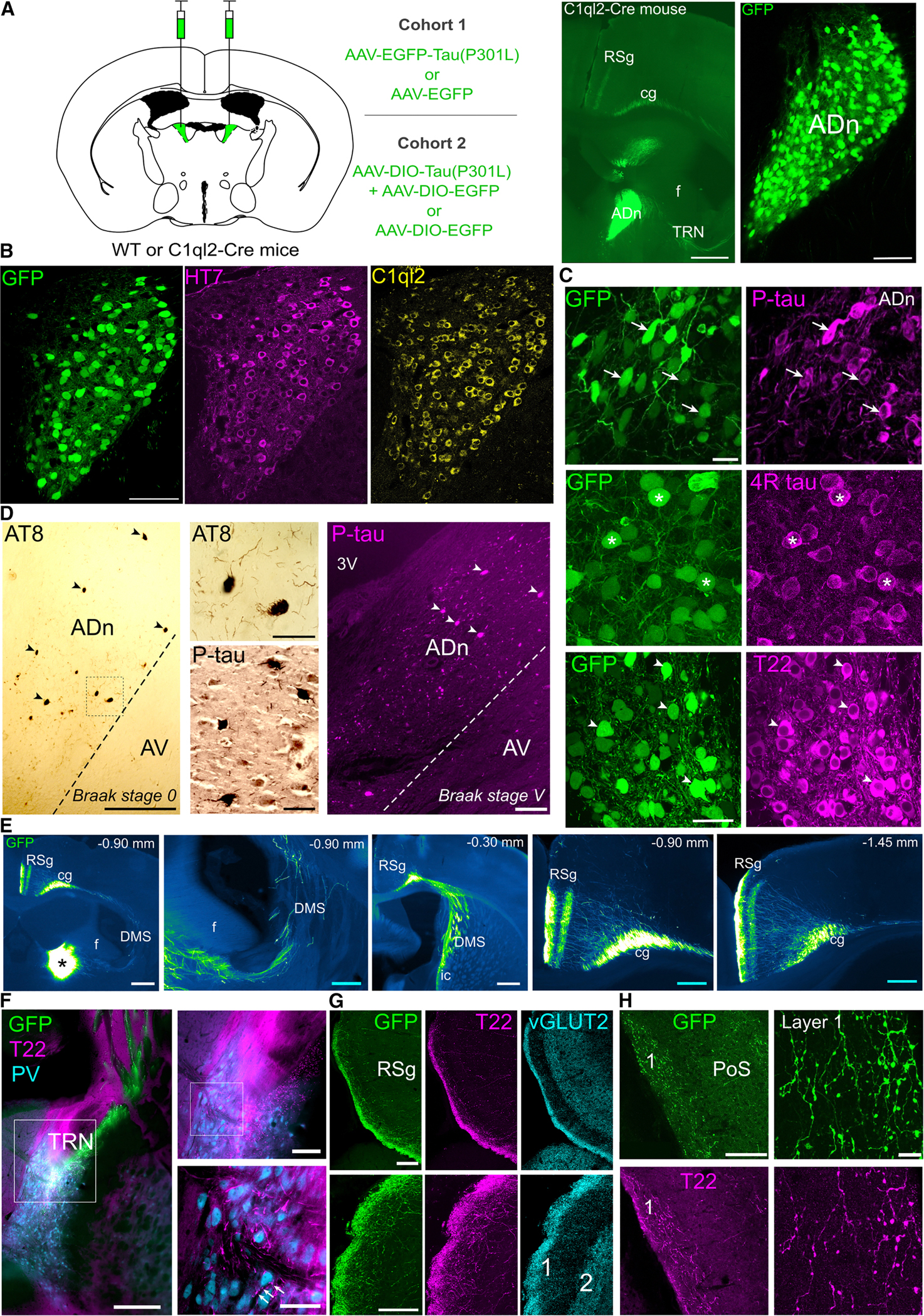
Defining ADn-tau mice by ptau in the HD network (A) Left: schematic of bilateral AAV injections into the ADn approximately −0.75 mm from the bregma. Cohort 1, WT mice; cohort 2, C1ql2-Cre mice. Middle and right: wide-field epifluorescence images showing GFP (green) localized to the ADn. Case SH68. (B) Confocal microscopic image of GFP (green) and human tau (HT7, magenta) colocalizing with C1ql2 (yellow) restricted to the ADn. Case SH71, single optical section. (C) Colocalization of GFP (green) with P-tau (top, magenta, arrows, case TV178), 4R Tau (middle, asterisks, case TV178), and T22 (bottom, arrowheads, case SH80) in the ADn. Confocal z projections (top, 29 μm thick; middle, 13.4 μm thick; bottom, single optical section). (D) Tau pathology in the human ADn. Left: AT8 immunoreactivity (horseradish peroxidase [HRP]-based diaminobenzidine [DAB] reaction) localized to the ADn, but not the AV, in a Braak stage 0 case. Bright-field image from a 5-μm-thick formalin-fixed paraffin-embedded (FFPE) section. Arrowheads, AT8+ somata. Middle top, detail of boxed region. Right, phospho-tau (P-tau; T231) immunoreactivity in the ADn of a Braak stage V case. Wide-field epifluorescence image from a 5-μm-thick FFPE section. Arrowheads, P-tau+ somata. Middle bottom, detail of P-tau+ somata (HRP-based DAB reaction, bright field). (E) Trajectory of GFP-expressing ADn cell axons to postsynaptic target regions (with approximate distance from the bregma indicated in each image). Left: injection site centered on the ADn (asterisk, brightness and contrast adjusted to reveal axons). Axons travel laterally and rostrally into the TRN, followed by the DMS, then cross the corpus callosum, entering the cingulum bundle (cg) and travel caudally to branch in the RSg. Wide-field epifluorescence micrographs, case SH30. (F) Left: axons from GFP-expressing ADn cells in the TRN and DMS. Inset top right: detail of T22+ axons (magenta) in the dorsal TRN surrounding PV-immunoreactive neurons (cyan). Inset bottom right: detail of innervated TRN cells (arrows). Wide-field epifluorescence micrographs, case SJ14. (G) Top: innervation of the RSg by GFP-expressing ADn axons from the ADn, with GFP (green) and T22 (magenta) localized to the vGLUT2-immunoreactive plexus (cyan) in layer 1. Case SH38, 28-μm-thick confocal maximum intensity z projection. Bottom: detail of ADn terminals in layer 1 of the RSg, 25.5-μm-thick confocal z projection. Case SH38. (H) Left: innervation of the PoS by GFP+ T22+ ADn axons, 20.6-μm-thick confocal z projection. Right: detail of ADn terminals in PoS layer 1, 25.9-μm-thick confocal maximum intensity z projection. Case SH38. Scale bars: (A) middle, 100 μm; (A) right, 20 μm; (B), 100 μm; (C), 20 μm; (D) left, 200 μm; (D) middle top, 25 μm, (D) middle bottom, 50 μm, (D) right, 100 μm; (E), 500 μm (white) and 250 μm (cyan); (F), 300, 100, and 50 μm; (G), 100 μm; (H) left, 100 μm, (G) right, 10 μm. sm, stria medullaris; f, fimbria; ic, internal capsule. See also Figure S1.

We bilaterally targeted the ADn of adult C57Bl6j (wild-type [WT]) mice with *AAV-CBh>EGFP:T2A:Tau(P301L):WPRE* (encoding GFP and mutant human tau) or a control adeno-associated virus (AAV) encoding GFP only (cohort 1; Figures 1A and S1A; Table S1). To increase specificity, we also injected C1ql2-Cre mice with Cre-dependent AAVs encoding mutant human tau and/or GFP, resulting in selective expression of ptau in C1ql2-expressing cells, which includes the majority of cells within the ADn and excludes all other thalamic nuclei (cohort 2; Figures 1A and S1A; Table S1). After at least 8 weeks of expression, we observed GFP fluorescence and/or ptau immunoreactivity in the HD network (Figures 1 and S1; Table S1; *n* = 33/37 injected mice). ADn-tau mice and ADn-gfp (control) mice were defined by GFP+ somata predominantly localized to the ADn (Figures 1A–1C and S1E–S1G) and GFP+ axon terminals in postsynaptic target regions of the ADn, including the thalamic reticular nucleus (TRN), DMS, RSg, and PoS (Figures 1E and S1D; Table S1).^30,31^

The ADn was identified by its high levels of lipofuscin and biotin (Figure S1B) as well as C1ql2 immunoreactivity, which was detectable in the majority of ADn neurons (Figure S1C).^42,47^ We detected ptau in ADn somata and dendrites in ADn-tau mice but not in ADn-gfp mice based on immunoreactivity to HT7 (human-specific tau), phospho-tau T231 (P-tau), 4R tau, and T22 (oligomeric tau) (Figures 1B, 1C, and S1E–S1G). We confirmed that ptau+ somata were detectable in postmortem human ADn (Figure 1D). Next, we quantified expression of ptau and GFP in both ADn-tau mice and ADn-gfp mice. The oligomeric, pre-aggregated form is considered a driver of early pathological changes.^48–50^ Approximately 90% of GFP+ cells in ADn-tau mice were T22+ (cohort 1: 90.5% ± 2.2%; cohort 2: 78.6% ± 3.9%; Figures 1C and S1E), whereas ADn-gfp mice predominantly lacked T22 immunoreactivity (cohort 1: 0% ± 0%; cohort 2: 3.5% ± 0.3%; Figure S1E). Mice had similar proportions of GFP+/C1ql2+ ADn cells (67.2% ± 1.2% GFP+/C1ql2+ cells in ADn-tau mice versus 60.6% ± 0.3% in ADn-gfp mice; *t* test, *t*_(*6*)_ = 0.5159, *p* = 0.6244; Figure S1F). We also assessed the extent of viral expression using semi-quantitative scoring (Table S1) and by quantifying T22 and NeuN immunoreactivity. We found that 68.4% ± 1.7% of NeuN+ cells were T22+ in ADn-tau mice (Figure S1G), similar to the proportion of GFP+/C1ql2+ cells (Figure S1F).

Axons and presynaptic terminals tested in the TRN, RSg, and PoS were immunoreactive for oligomeric tau in ADn-tau mice but not in ADn-gfp mice (Figures 1F–1H, S1H, and S1I). The ptau+ axons in postsynaptic target regions were GFP+ (Figures 1G and 1H). We did not detect ptau+ somata in postsynaptic regions of the ADn, suggesting a lack of transsynaptic spread for at least 27 weeks.^51^ In the TRN, ADn axons formed basket-like terminals around parvalbumin (PV)-expressing neurons (Figure 1F). In the RSg, axon terminals formed a plexus in the superficial part of layer 1 and colocalized with vesicular glutamate transporter 2 (vGLUT2; Figure 1G). A similar pattern was observed in the PoS (Figure 1H). These innervation patterns are consistent with the pattern of ptau immunoreactivity detected in the human ADn, TRN, and RSg at early Braak stages.^13^

### Disorientation-related alterations of spatial navigation in ADn-tau mice

**Figure 2.**
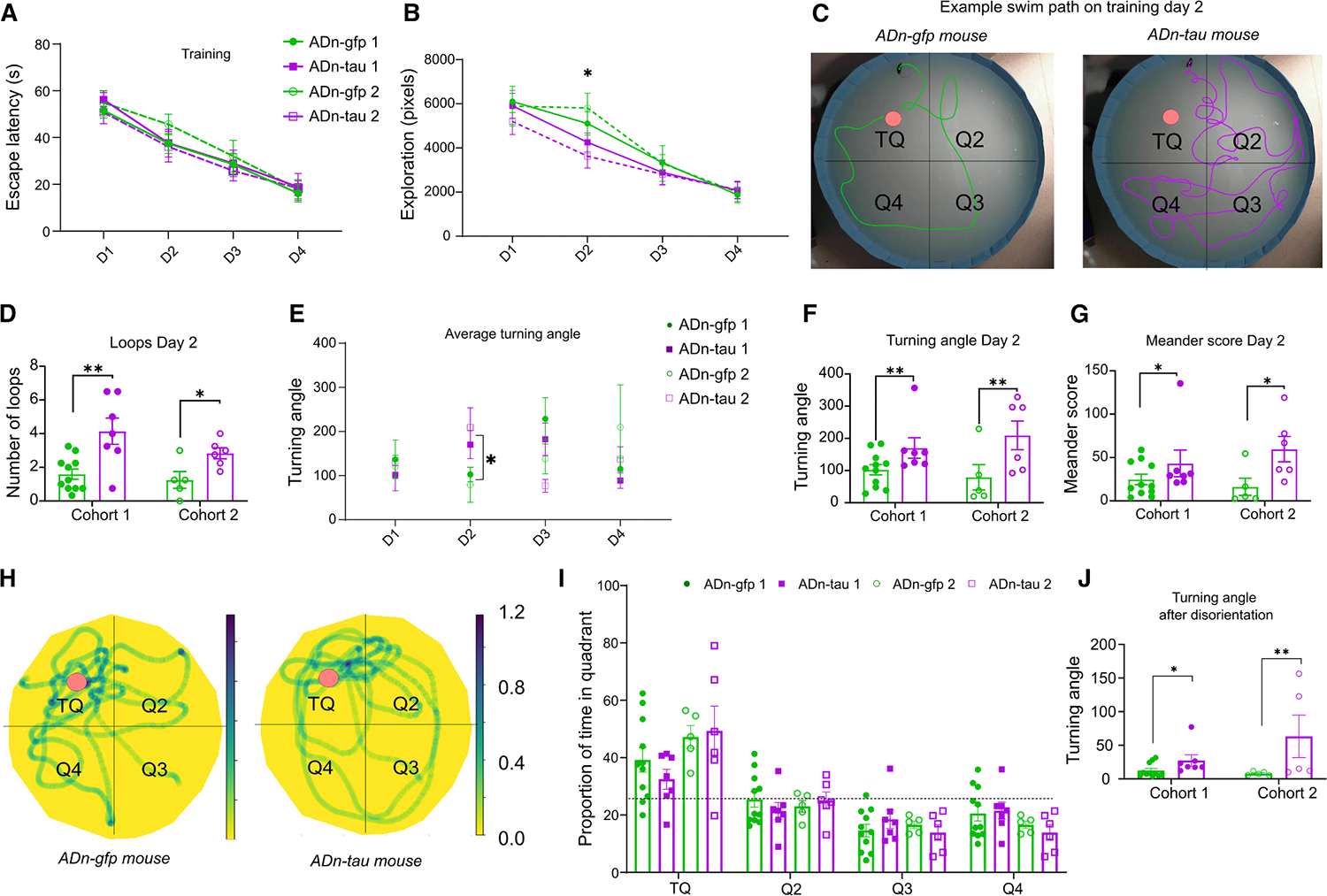
ADn-tau mice exhibit disorientation during a spatial reference memory task (A) Escape latency in the MWM over 4 consecutive training days (days 1–4) for cohorts 1 and 2. (B) Quantification of individual exploration (coverage) of the MWM for days 1–4. (C) Example images of swim paths for an ADn-gfp mouse (left, green) and an ADn-tau mouse (right, purple) on day 2. Target quadrant, TQ; other quadrants, Q2–Q4. (D) Average number of loops on day 2. (E) Quantification of average turning angle for days 1–4. (F) Turning angle on day 2. (G) Meander score on day 2. (H) Example heatmaps of swimming activity during the probe test. Scale bar: occupancy in seconds. (I) Quantification of time in each quadrant. Chance level: 25% (dashed line). (J) Quantification of turning angle after inducing disorientation. Data are represented as mean ± SEM; *p < 0.05, **p < 0.01. See also Figure S2 and Videos S1, S2, S3, S4, S5, S6, and S7.

**Figure 3.**
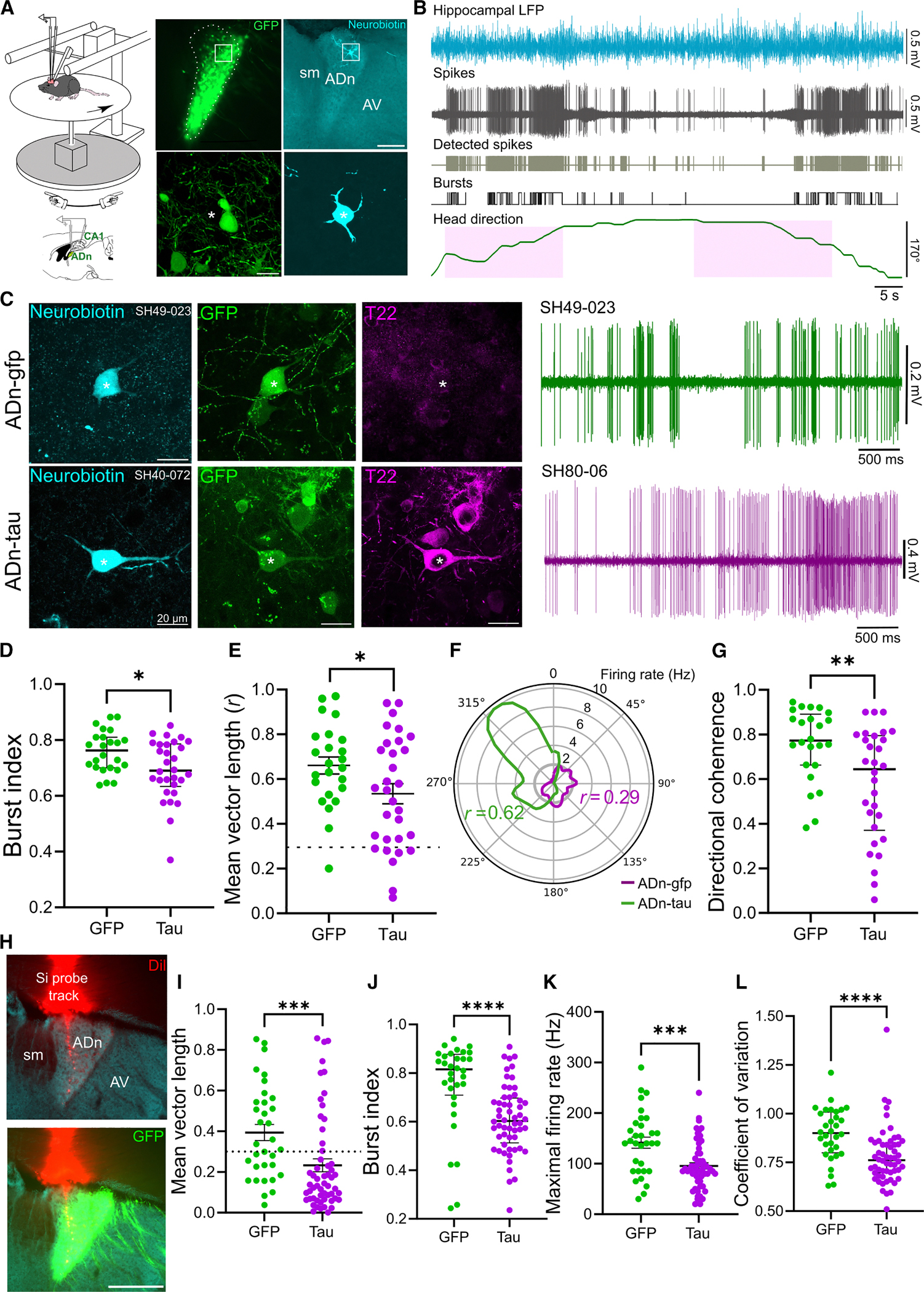
Reduced directionality of ADn cells from ADn-tau mice (A) Top left: schematic of the recording setup. The turntable (gray) is passively rotated. The mouse is able to spontaneously run and rest on a running disc. Bottom left: sagittal brain section indicating recording locations. Top right: epifluorescence micrograph of a coronal brain section showing viral expression of GFP (green) in the ADn and a neurobiotin-labeled HD cell (cyan, cell SH79–07); scale bar, 100 μm. Bottom right: enlarged view of the labeled cell, confocal maximum intensity z projection (7.6 μm thick); scale bar, 20 μm. (B) Simultaneous recording of the hippocampal (CA1) LFP (cyan), action potentials of an ADn HD cell (SH30–05, black), detected bursts (gray), and HD (green). The shaded purple area marks the preferred firing direction. (C) Top left: a juxtacellularly labeled ADn cell (neurobiotin, cyan, SH49–023, asterisk) that expressed GFP (green) but lacked detectable immunoreactivity for T22 (magenta). Confocal maximum intensity z projection (11.7 μm thick). Bottom left: a juxtacellularly labeled ADn cell (SH40–072, asterisk) that expressed GFP was immunopositive for T22. Confocal maximum intensity z projection (5.1 μm thick). Scale bars, 20 μm. Right: firing patterns of a GFP+ T22− ADn cell SH49–023 and a GFP+ T22+ ADn cell (SH80–06). (D–G) Electrophysiological properties of ADn cells recorded with glass electrodes (D, burst index; E and F, mean vector length; G, directional coherence) from ADn-gfp mice (green) and ADn-tau mice (purple). Dashed line, threshold for non-HD (NHD) cells (*r* < 0.3). (F) shows a polar plot of representative tuning curves (ADn-tau mouse, cell SH40–072, purple; ADn-gfp mouse, cell TV190–2i, green). (H) Wide-field fluorescence micrograph of a coronal brain section showing viral expression of GFP (green) and a probe tract (DiI, red) localized to the ADn, case SH73. Scale bar, 300 μm. (I–L) Electrophysiological properties of ADn cells recorded with silicon probes (I, mean vector length; J, burst index; K, maximal firing rate; L, coefficient of variation) from ADn-gfp mice (green) and ADn-tau mice (purple). Data are represented as mean ± SEM (E), (I,) (K) or median and IQR (D), (G), (J), (L);*p < 0.05, **p < 0.01, ***p < 0.001, ****p < 0.0001. See also Figures S3 and S4.

**Figure 4.**
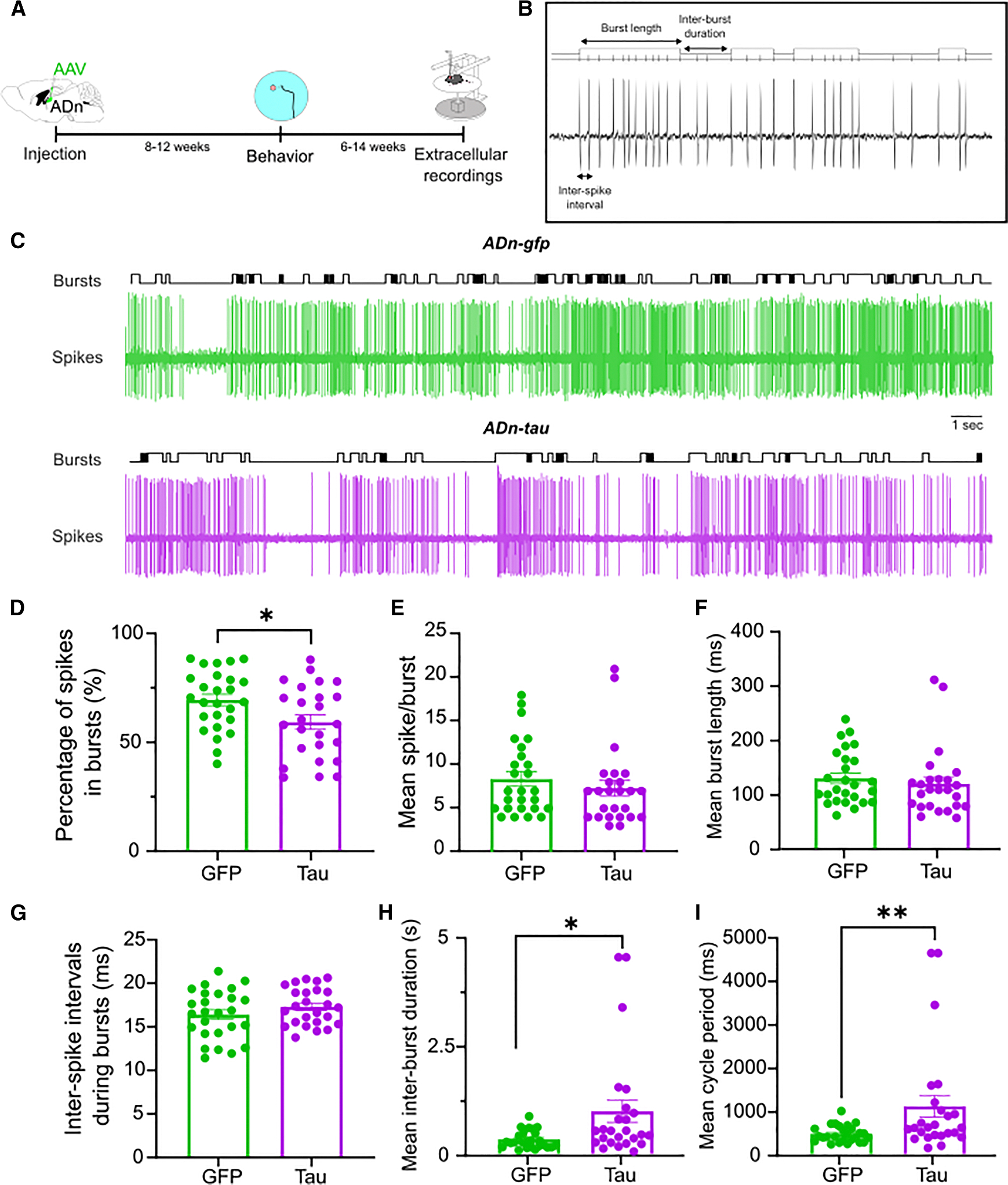
Altered burst firing of HD cells from ADn-tau mice (A) Schematic of the timeline. (B) Parameters for the burst analysis. (C) Example of burst firing patterns from two HD cells: top, ADn-gfp mouse (SH30–1-h); bottom, ADn-tau mouse (SH33–10). (D) Percentage of spikes during bursts for HD cells. (E) Mean number of spikes per burst (*t*_(49)_ = 0.8634, *p* = 0.3921). (F) Mean duration of bursts (*t*_(49)_ = 1.283, *p* = 0.2054). (G) Inter-spike interval during bursts (*U* = 264, *p* = 0.2547). (H) Mean duration between bursts. (I) Average burst cycle period. Data are represented as mean ± SEM; *p < 0.05, **p < 0.01.

Spatial navigation is dependent on the HD network.^36,38,52,53^ To establish whether ptau in the HD network affects spatial navigation and orientation, we trained mice in spatial reference and spatial working memory tasks after 8–12 weeks of viral expression (Figures 2 and S2; Table S1). This was followed by *in vivo* recordings in the behaviorally tested mice (Figures 3, 4A, and S3).

Spatial reference memory was assessed using the Morris water maze (MWM), where mice had to learn to find a hidden escape platform using allocentric, extramaze spatial cues (Figures 2A–2G and S2A–H). This was followed by a probe test, where the platform was removed and mice explored the MWM for 60 s (Figures 2H and 2I). The ADn-tau and ADn-gfp groups showed similar latencies when locating the platform during training (training effect [main effect of day] two-way repeated measures ANOVA: cohort 1, *n* = 7 ADn-tau mice and *n* = 11 ADn-gfp mice, *F*_3,48_ = 37.57, ***p < 0.0001; cohort 2, *n* = 6 ADn-tau mice and *n* = 5 ADn-gfp mice, *F*_3,27_ = 27.15, ***p < 0.0001; Figure 2A), and no differences were detected across groups (group effect two-way repeated measures ANOVA: cohort 1, *F*_1,16_ = 0.1652, *p* = 0.6898; cohort 2, *F*_1,9_ = 0.9225, *p* = 0.3619; group × training effect two-way repeated measures ANOVA: cohort 1, *F*_3,48_ = 0.1763, *p* = 0.911; cohort 2, *F*_3,27_ = 0.5159, *p* = 0.675; Figure 2A). As the ADn contains a high density of HD cells, we hypothesized that ADn-tau mice would show differences in how they orient themselves in the maze during training.

To assess spatial orientation during learning, we examined swimming patterns across the 4 training days. We observed that ADn-tau mice explored the maze less than ADn-gfp mice on training day 2 (group effect two-way repeated measures ANOVA: *F*_1,27_ = 26.25, *p < 0.05; nested *t* test for day 2, *t*_*(27)*_ = 2.564, *p < 0.05; Figure 2B). During day 2, we observed ADn-tau mice making several localized loops, mostly in the same direction (clockwise or counterclockwise), while searching for the platform (Figure 2C; Videos S1, S2, S3, S4, and S5). We found that ADn-tau mice made a significantly greater number of loops across the four trials on day 2 compared to ADn-gfp controls (cohort 1, median [interquartile range (IQR)] 4 [3–6.5] loops for ADn-tau mice versus 1.25 [0.75–2.25] loops for ADn-gfp mice; *U* = 10.5, **p = 0.0093; cohort 2, 2.85 [1.75–4] loops for ADn-tau mice versus 1.25 [0–3] loops for ADn-gfp mice; *U* = 3.5, *p = 0.0390; Figures 2D and S2B; Videos S1, S2, S3, S4, and S5).

In agreement with the increased number of loops, ADn-tau mice made significantly more turns (quantified as turning angle) when exploring the maze on day 2 compared to ADn-gfp mice, which we independently observed in both cohorts (group × training effect two-way repeated measures ANOVA: *F*_3,27_ = 26.25, *p < 0.05, post hoc Fisher’s least-significant difference (LSD) test for day 2: *p < 0.05; Figure 2E; turning angle on day 2: cohort 1, *U* = 19, *p < 0.05; cohort 2, *t*_(*9*)_ = 2.145, *p < 0.05; Figure 2F). This was also evident in their average meander score (an estimation of their path deviation during swimming) on day 2 (cohort 1, *U* = 3, *p < 0.05; cohort 2, *t*_(*9*)_ = 2.348, *p < 0.05; Figure 2G). These data demonstrate that ADn-tau mice conducted more turns during their path to the platform during the second training day. Interestingly, the average turning angle positively correlated with GFP expression in ADn-tau mice (Spearman *r* = 0.688, *p < 0.05) but not in ADn-gfp mice (Spearman *r* = 0.029, *p* = 0.929; Figure S2C).

What could explain the similar latencies to the hidden platform on day 2 (Figure 2A), given the difference in meander score (Figure S2D)? ADn-tau mice had shorter path lengths to the hidden platform on day 2 (cohort 1, *t*_(*16*)_ = 2.296, *p = 0.035; cohort 2, *t*_(*9*)_ = 2.607, *p = 0.028; Figure S2E) with a slower swimming speed (cohort 1, *t*_(*16*)_ = 2.129, *p = 0.049; cohort 2: *t*_(*9*)_ = 2.756, *p = 0.022; Figure S2F), explaining why ADn-tau mice covered, on average, a smaller exploration area within the same time compared to ADn-gfp mice (Figure 2A). There was no significant difference in the average path length in relation to days of training (training × group effect two-way repeated measures ANOVA: *F*_1,27_ = 1.287, *p* = 0.2581; Figure S2G) or in the overall swimming speed (training × group effect two-way repeated measures ANOVA: *F*_1,27_ = 0827, *p* = 0.593; Figure S2H). Accordingly, the increased looping was resolved by day 3, suggesting that ADn-tau mice have altered orienting responses only during the initial acquisition of the spatial map (Figures S2B–S2H).

In the probe test, both groups spent more time in the target quadrant (TQ), which previously contained the hidden platform, compared to chance, and there were no differences between groups (cohort 1: ADn-gfp versus chance, *U* = 7, ***p < 0.001; ADn-tau versus chance, *U* = 7, *p < 0.05; ADn-gfp versus ADn-tau, *t*_(*16*)_ = 0.770, *p* = 0.4522; cohort 2: ADn-gfp versus chance, *U* = 0, **p < 0.01; ADn-tau versus chance, *U* = 6, *p < 0.05; ADn-gfp versus ADn-tau, *t*_(9)_ = 0.208 *p* = 0.839; Figures 2H and 2I; Table S2), indicating that both groups of mice learned the location of the platform. No differences were detected for the number of entries, total path length, or swim speed in the TQ (Table S2).

To test whether the increased looping was linked to alterations in spatial orientation, we challenged mice by testing them in the MWW immediately after disorienting them. This was achieved by placing each mouse in a small opaque box, rotating the box several times, then immediately placing them in the MWM (Figure S2A; STAR Methods). Both groups spent a similar proportion of time in the TQ, remaining above chance (cohort 1: ADn-gfp versus chance: *U* = 0, **p < 0.01; ADn-tau versus chance: *U* = 0, **p < 0.01; cohort 2: ADn-gfp versus chance: *U* = 0, **p < 0.01; ADn-tau versus chance: *U* = 0, **p < 0.01; Figure S2I). However, ADn-tau mice exhibited an increased average turning angle compared to ADn-gfp mice following disorientation (cohort 1: *U* = 17, *p < 0.05; cohort 2: *U* = 1, **p < 0.01; Figure 2J; Videos S6 and S7).

To confirm that the increased turning was a result of disorientation rather than spatial memory deficits, we tested mice in a reversal learning protocol, where the platform location was swapped to the opposite quadrant (new TQ). We detected no differences in performance between groups for the training trials or in post-reversal probe tests (Figure S2J; Table S2). ADn-tau mice did not exhibit disorientation during training, based on the low number of loops, and had exploration, meander scores, and average turning angles that were similar to ADn-gfp mice (Table S3). Finally, we performed a long-term spatial reference memory test by conducting another probe test 3 weeks later (Figure S2A) and found that both groups spent a significantly longer time in the TQ (Figure S2K; Table S2). These results suggest unimpaired spatial reference memory.

Spatial working memory was tested using a rewarded alternation non-matching-to-place T-maze task (Figure S2L). Both groups learned the task (training effect [main effect of day] two-way repeated measures ANOVA: cohort 1, *n* = 7 ADn-tau mice and *n* = 11 ADn-gfp mice, *F*_2,32_ = 3.342, *p = 0.0151; cohort 2, *n* = 6 ADn-tau mice and *n* = 5 ADn-gfp mice, *F*_4,36_ = 6.917, ***p = 0.0003; Figure S2M). We also did not detect any differences in terms of locomotor activity or anxiety-related behavior (Figure S2N). Mice were also tested in a spatial novelty preference task.^54^ Both groups showed an equivalent preference for the novel arm (Figure S2O).

Taken together, these data suggest that ADn-tau mice represent an early disorientation model where mice develop no apparent spatial memory impairments yet show specific differences in their orienting responses during the acquisition of the spatial map.

### Reduced directionality of ADn cells in ADn-tau mice

We hypothesized that the differences in the orienting response during spatial reference memory acquisition in ADn-tau mice were associated with ptau affecting ADn HD cells. To test this, we targeted the ADn with glass electrodes in the previously behaviorally tested mice. To detect HD cells, we passively rotated head-restrained mice on a turntable with respect to fixed visual cues (Figure 3A), which provides the necessary rotational vestibular and visual inputs.^55,56^ Following single-neuron extracellular recordings (Figure 3B), we juxtacellularly labeled selected cells with neurobiotin for post hoc recovery and confirmation of recording locations (Figures 3A and S3A), thus providing a robust classification of ADn cells with respect to GFP expression and ptau immunoreactivity (Figures 3A–3C and S3B).

We learned to recognize the ADn by the presence of HD cells that exhibited irregular burst firing and a lack of coupling to hippocampal theta oscillations (Figures 3B, S3D, and S3E).^16^ We localized 116 single extracellularly recorded cells to the ADn based on the locations of 40 recovered juxtacellularly labeled cells from 16 mice (Table S1; Figure S3A). We excluded 61 ADn cells due to a lack of GFP/ptau in the recording locations, resulting in 31 and 24 ADn cells from ADn-tau and ADn-gfp mice, respectively. Among the labeled cells, 3 cells expressed GFP, with 2 cells from ADn-tau mice showing immunoreactivity to T22 (Figures 3C and S3B). Examination of their firing patterns in their preferred firing directions suggested differences in bursting activity between the ptau− and ptau+ cells (Figures 3C and 4). Indeed, cells from ADn-tau mice had a lower burst index than those from ADn-gfp mice (0.69 [0.64–0.78] from ADn-tau mice versus 0.76 [0.7–0.81] from ADn-gfp mice; *U* = 246, *p = 0.03; Figure 3D).

Next we compared the strength of the directional signal (also known as directionality, indicated by mean vector length *r*), and found that ADn cells from ADn-tau mice had lower directionality (mean ± SEM, *r* = 0.53 ± 0.04 from ADn-tau mice versus *r* = 0.66 ± 0.04 from ADn-gfp mice; *t*_(53)_ = 2.079, *p = 0.0425; Figures 3E and 3F) and lower directional coherence (0.66 [0.38–0.8] from ADn-tau mice versus 0.77 [0.66–0.89] from ADn-gfp mice; *U* = 220, **p = 0.009; Figure 3G). These results suggest that, on average, ADn cells from ADn-tau mice have reduced directional coding with jagged irregular tuning curves.

We used a threshold of *r* = 0.3 to separate ADn HD cells from non-HD (NHD) cells based on published data from the mouse ATN.^55^ We observed a slightly lower proportion of ADn HD cells in ADn-tau mice versus ADn-gfp mice (80.7% versus 95.8%; Fisher’s exact test, *p* = 0.09). Within the subpopulation of ADn HD cells (*r* > 0.3), we found that ptau did not significantly alter directionality (*r* = 0.61 ± 0.04 for 20 cells from ADn-tau mice versus 0.68 ± 0.03 for 23 cells from ADn-gfp mice; *t*_(41)_ = 1.251, *p* = 0.5305; Figure S3H). The peak and background firing rates were similar (Figures S3I and S3J), as were the directional tuning widths (103.4° [73.54°–225°] from ADn-tau mice versus 106.9° [60.8°–180°] from ADn-gfp mice; *U* = 215, *p* = 0.72; Figure S3K). The distribution of preferred firing directions was also similar in both groups (Figure S3L). This indicates that, in ADn-tau mice, some HD cells remain unaffected, whereas others have greatly reduced directionality, which may reclassify them as NHD cells.

Following glass electrode recordings, we lowered a silicon probe to the same location to record at the population level (Figures 3H–3L and S3M–S3T). Consistent with the single-cell recordings, we observed lower directionality within ptau-enriched zones compared to control GFP-enriched zones (*r* = 0.14 [0.07–0.29] for 56 units from 3 ADn-tau mice versus *r* = 0.34 [0.21–0.55] for 33 units from 3 ADn-gfp mice; *U* = 494, ***p = 0.00023; Figure 3I; Table S1) and a lower proportion of ADn HD cells in ADn-tau mice versus ADn-gfp mice (25% versus 43.8%; Fisher’s exact test, *p* = 0.09), further suggesting that there were fewer HD cells in ADn-tau mice. Similarly, we also observed a lower burst index (0.6 [0.51–0.9] from ADn-tau mice versus 0.82 [0.71–0.94] from ADn-gfp mice; *U* = 397.5, ****p < 0.0001; Figure 3J). We additionally observed a lower maximal firing rate (90 [67.5–240] Hz from ADn-tau mice versus 140 [85–178] Hz from ADn-gfp mice; *U* = 515, ***p < 0.001; Figure 3K) and a lower coefficient of variance (CV; 0.76 [0.7–0.85] from ADn-tau mice versus 0.89 [0.79–1] from ADn-gfp mice; *U* = 441, ****p < 0.0001; Figure 3L). Overall, the silicon probe data were in agreement with the single-neuron recordings.

When examining the firing properties of NHD cells in the ADn, we found that NHD cells from ADn-tau mice had, on average, lower mean vector lengths compared to those from ADn-gfp mice (*r* = 0.11 ± 0.01 from ADn-tau mice, *n* = 42 units, versus *r* = 0.18 ± 0.02 from ADn-gfp mice, *n* = 18 units; *t*_(58)_ = 3.754, ***p = 0.0006; Figure S3Q). The burst index (0.6 ± 0.02 from ADn-tau mice versus 0.77 ± 0.03 from ADn-gfp mice; *t*_(44)_ = 4.325, ****p < 0.0001; Figure S3R), CV (0.78 ± 0.02 from ADn-tau mice versus 0.93 ± 0.03 from ADn-gfp mice; *t*_(58)_ = 4.116, ***p = 0.0001; Figure S3S), and maximal firing rates (90 [70–106.3] Hz from ADn-tau mice versus 147.5 [81.25–190] Hz from ADn-gfp mice; *U* = 209, **p = 0.0056; Figure 3T) were also lower in ADn-tau mice compared to ADn-gfp mice, suggesting that NHD cells are likely to be ptau-expressing ADn cells. Of note, viral expression did not correlate with mean vector length for either group (Spearman *r* = −0.22, *p* = 0.2458 for ADn-tau mice versus *r* = −0.3, *p* = 0.1548 for ADn-gfp mice; Figure S3C; Table S1).

### Altered burst firing of HD cells in ADn-tau mice

Given the reduced burst indices in ADn-tau mice (Figures 3D and 3J), we further investigated the differences in firing within the HD cell receptive fields. We observed that HD cells from ADn-tau mice had a lower percentage of spikes per burst (*n* = 25 cells from ADn-tau mice versus 26 cells from ADn-gfp mice; *t*_(49)_ = 2.378, *p = 0.0214; Figures 4C and 4D), an increased duration between bursts (*U* = 198, *p = 0.0161; Figure 4H), and a longer average burst cycle period (*U* = 186, **p = 0.0082; Figure 4I). This is consistent with the altered bursting patterns of HD cells following conditions that promote disorientation via prolonged rotation,^57^ suggesting that ADn-tau mice exhibited disorientation due to altered burst firing patterns. No significant differences were observed in the number of spikes per burst (Figure 4E), mean burst durations (Figure 4F), or inter-spike intervals during bursts (Figure 4G). Finally, we examined theta and gamma oscillations in hippocampal CA1 (Figure S4A), which are indicators of spatial memory-related neuronal coordination. We did not detect any significant differences in instantaneous frequency or amplitude between ADn-gfp and ADn-tau mice (Figures S4B–S4F), suggesting that neuronal coordination is unimpaired in the hippocampus of ADn-tau mice. This is consistent with the lack of viral expression in CA1 (Figure S1D; Table S1).

Taken together, these data suggest that ptau selectively affects the directionality of ADn cells and the firing mode of HD cells within the preferred firing direction. These disrupted firing properties may explain the markedly different orienting responses during spatial learning in the MWM.

## DISCUSSION

Spatial disorientation is emerging as an early cognitive biomarker for dementia,^8^ which might be explained by impairments in the HD network.^7^ The human ADn contains a high density of ptau,^14,15,58^ which is even detectable at Braak stage 0.^13^ This may affect the function of HD cells and, in turn, synaptic integration by postsynaptic target neurons. To this end, we virally expressed mutant human tau in the ADn, resulting in oligomeric and phosphorylated forms of ptau accumulating in somata, dendrites, and axons of ADn cells. Axon terminals containing ptau were distributed in postsynaptic target areas, including the TRN, DMS, RSg, and PoS.

Our data reveal that, while ADn-tau mice could learn spatial reference and spatial working memory tasks, which are well-known hippocampus-dependent forms of spatial memory,^59,60^ they used a different exploration strategy (a change in their orienting responses), expressed as increases in looping, or turning angle and meander score, on day 2 of training in the MWM. This occurred in two independent cohorts the day after they were first exposed to the location of the hidden platform. We attribute this to the effect of ptau on the HD network “upstream” of the (unimpaired) hippocampus. By recording in the ADn, we found that ADn cells from ADn-tau mice had significantly reduced directionality and lower directional coherence. The remaining HD cells also exhibited altered burst firing. These alterations likely affected the coordination of postsynaptic target neurons, such as cortical HD cells,^61^ promoting spatial disorientation.

### Pathological tau contributes to spatial disorientation during memory acquisition

The ADn has been characterized as a key brain region for spatial navigation,^39,62^ but to fully understand its role in spatial memory, it needs to be distinguished from the other anterior thalamic nuclei. Selective optogenetic inhibition of the ADn impairs spatial working memory.^40,42^ Note that this is different from our approach, as we did not silence ADn neurons but, rather, expressed human tau in them. An increasing body of evidence now positions the ADn as a critical component in the broader neural circuitry underlying spatial navigation and memory, with the ADn required for integrating both local and distal visual cues.^63,64^ These findings underscore the ADn not only as a relay of directional information but also as a potential site for integrative computations essential for maintaining coherent spatial representations.^65,66^ Given the functional significance of ADn HD cells and their connections with parahippocampal areas, it becomes increasingly important to investigate whether alterations of ADn HD and NHD cells (rather than lesions or silencing) can directly impact navigational abilities.

Castegnaro et al.^7^ employed a virtual reality navigation task in humans, finding that individuals with MCI could be distinguished from controls based on both direction and distance traveled. Similarly, Hanyu et al.^67^ demonstrated that impairments in path integration could be used to detect prodromal Alzheimer’s disease and predict cognitive decline over a 12-month period. Prior studies have shown that path integration deficits are prevalent in patients with amnestic MCI.^68,69^ Given that path integration tasks are simple, non-invasive, and cost effective, they may serve as promising clinical tools for early screening and therapeutic stratification in individuals at risk of Alzheimer’s disease. Therefore, understanding the cellular and circuit-level mechanisms—particularly those involving HD cells—that underlie such early spatial navigation deficits is of high translational relevance.

How does the accumulation of ptau in ADn cells affect spatial learning? We observed marked differences in the MWM on training day 2, when ADn-tau mice explored the maze differently to reach the hidden platform, exhibiting looping behavior, in contrast to GFP-expressing control mice. This was striking, as the effect on day 2 was observed in two separate cohorts. Looping is reminiscent of spatial disorientation, as suggested by other studies.^57,63^ Indeed, mice that are disoriented immediately before testing in an MWM exhibit looping behavior, are less likely to use spatial strategies to find the hidden platform, and make more heading errors.^63^ It is noteworthy that, in our experiments, ADn-tau mice showed increased looping even without disorienting them prior to the MWM. These observations suggest an impairment in the early but not late phases of spatial memory acquisition.

Donato et al.^70^ showed that, in the initial phase of MWM training, hippocampal neurons undergo structural and network-related plasticity that is required for spatial memory acquisition and that this plasticity is at its highest on day 2. Another study found that hippocampal place cell firing rates and the accuracy of spatial information increase dramatically between days 1 and 2 of incremental spatial learning.^71^ This suggests that ptau alters communication between brain regions required for spatial navigation in ADn-tau mice at this critical time despite mice being able to successfully acquire this form of spatial reference memory. A likely source of this interference is the RSg, which receives extensive input from the ADn,^31,61^ is required for spatial learning,^72^ and, like the ADn, is affected by ptau at early Braak stages.^13^

### Effects of ptau on HD cell activity explains disorientation

Directional information is crucial for spatial navigation,^73,74^ and manipulating the HD network can affect this process. For example, chemogenetic inhibition of the anterodorsal TRN broadens the directional tuning of ADn HD cells and alters search strategies in the MWM.^75^ Grieves et al.^57^ showed that disorientation, which is observed as looping behavior, causes a decrease in the directionality, peak firing rate, and burst index score of ADn HD cells. This suggests that the altered orienting responses experienced by ADn-tau mice are due to reduced directionality of ADn cells as well as their altered burst firing patterns. This may affect path integration in other behavioral tasks.^45^

Through what mechanisms might ptau affect the activity of ADn cells? Previous work in animal models has shown that ptau can disrupt neuronal and synaptic function. Hyperphosphorylated tau reduces neuronal excitability, alters neuronal oscillatory patterns, prolongs cortical down states, and decreases firing rates.^76–83^ Furthermore, ptau can depolarize the resting membrane potential and increase the depolarizing voltage deflection or “sag” evoked by hyperpolarization. The increased sag potential has been linked to disruptions in dendritic microtubule-dependent transport of hyperpolarization-activated cyclic nucleotide-gated channels.^84^ Tau secreted into human cerebrospinal fluid induces neuronal hyperexcitability,^85^ and tau oligomers interfere with action potential waveforms, increasing firing rates.^86,87^ Future studies are needed to determine whether HD and NHD cells in ADn-tau mice have altered excitability and whether reducing ptau levels rescues HD cell firing and “normalizes” the orienting response during spatial learning.

Firing patterns of HD cells, including burst firing, have been suggested to support both the precision and persistence required for updating the directional heading. A malformation of the horizontal semicircular canals can induce bursting in the ADn, leading to an unstable HD signal.^88^ Inputs from the ATN, CA1 and dorsal subiculum converge on layer 5 RSg pyramidal neurons.^89^ Therefore, alterations of the burst firing of ADn HD cells in ADn-tau mice potentially alters the spatial and temporal summation of cortical and thalamic inputs by RSg neurons, which, we predict, would affect synaptic integration in RSg HD cells and may also impact HD cell-dependent encoding of allocentric boundary cells and grid cell firing.^26,90–92^ Simonnet and Brecht^93^ found that bursting activity of subicular principal cells correlates with encoding of spatial information, revealing that bursts induce sharper firing fields and convey more spatial information than regular spikes. Burst firing has also been found to affect the degree of cortical responsiveness.^94,95^ Taken together, burst firing has been shown to be functionally relevant to spatially tuned neurons, possibly by serving as a mechanism to transmit spatial information to downstream structures.

In another study that reported spatial memory deficits following ATN lesions, the authors detected decreased bursting in cells of the subiculum, a known postsynaptic region of the ATN, with a decrease in the average spikes per burst and an increase in the inter-burst interval.^38^ Inactivation or lesions of the ATN have been shown to significantly compromise grid cell function in the entorhinal cortex, emphasizing the interdependence of these spatial navigation systems.^65^ Accordingly, the lower directionality of ADn cells in ADn-tau mice implies that ptau might have downstream effects, leading to impaired firing in postsynaptic target regions.

### Vulnerability of the HD network

What makes the ADn so vulnerable to tau pathology and neurodegeneration?^14,15^ And why specifically the ADn and not the entire ATN? We propose that this is due to a combination of persistently high firing rates, maintenance of large axonal arbors, and high rates of autophagy. First, compared to the cortex, the ADn contains a high density of HD cells, which fire at high rates within the preferred firing direction,^16^ with little accommodation or adaptation.^96^ We hypothesize that, when the head is facing the same direction for a prolonged time period, the subpopulations of HD cells in the ADn for this preferred firing direction would need to maintain high firing rates.^46,97^ A change in head position would presumably shift this demand to other cells. The very high levels of biotin we observe in the mouse ADn, in contrast to the AV (Figure S1), are consistent with other neurons that maintain high rates (such as “fast-spiking” GABAergic neurons).^98^

Second, large axonal arbors with hundreds of thousands of presynaptic terminals would place high metabolic demands on a cell. This is the case for the dopaminergic neurons of the substantia nigra, which have enormous axonal arbors and are highly vulnerable to the accumulation of aggregates of alpha-synuclein, leading to neurodegeneration in Parkinson’s disease.^99^ Although no studies have reported full single-neuron reconstructions of ADn cells that include the presynaptic terminals (to our knowledge), we predict that individual ADn cells have extensive and multiply branching axons, which is supported by the observation of multiple target regions and the dense plexus of small terminals in the superficial RSg and PoS.

Third, neurons with a high energy demand require high rates of clearance of metabolic waste products via autophagy, with the most vulnerable neurons having the highest axon-to-soma cytoplasmic volume ratio.^100^ We observed high levels of lipofuscin in the mouse ADn compared to adjacent regions, suggesting a demand on the autophagy-lysosomal pathway, which may also include degradation of ptau retrogradely transported from axon terminals. The AV, which also projects to layer 1 of the RSg,^32^ has a lower density of HD cells that are distributed mainly in the medial part of the AV closest to the ADn.^101,102^ The human AV is relatively resistant to neurodegeneration and accumulates ptau only in late Braak stages.^13,14^ The high metabolic demand of ADn cells may contribute to the observation that the retrosplenial cortex and cingulum bundle are the earliest sites to exhibit glucose hypometabolism in early Alzheimer’s disease.^103,104^

### Limitations of the study

The majority of tauopathy animal models exhibit wide-ranging cortical pathology, making it difficult to confidently link any detectable cognitive impairment to a particular brain region or cell type.^105^ To overcome the limitations of transgenic lines, we expressed mutant human tau in the ADn, which was more precise in cohort 2. While our data focused on the ADn as a hub within the HD network, other regions containing spatially responsive cells, such as grid cells or border cells, may also be affected by ptau expression.^106^ Future studies investigating the firing properties of postsynaptic neurons in the RSg, PoS, or entorhinal cortex are required to determine the effects on the entire network. We propose that ADn-tau mice represent a model of early spatial disorientation during early Braak stages, where disoriented mice are distinguished from healthy controls by the alternations in exploration and orienting behavior when initially forming a spatial map. Further studies are required to investigate whether age, the most common risk factor of Alzheimer’s disease, in combination with prolonged ptau expression would induce spatial memory deficits in the longer term.

In conclusion, this study demonstrates that accumulation of ptau in the HD network reduces the directionality of ADn cells and alters the firing patterns of HD cells, which, we suggest, promotes disorientation, affecting the way in which mice acquire a spatial map during initial learning. Subtle behavioral changes in spatial navigation and orientation in aged individuals may serve as a compelling early cognitive biomarker for predicting MCI or dementia. Earlier detection of at-risk individuals could enhance opportunities for intervention through dietary modifications or other lifestyle changes as well as early pharmacological and immunological treatment strategies.

## RESOURCE AVAILABILITY

### Lead contact

Requests for further information, resources, and reagents should be directed to and will be fulfilled by the lead contact, Tim J. Viney (tim.viney@pharm.ox.ac.uk).

### Materials availability

Apart from the viral vectors (key resources table), this study did not generate new unique reagents.

### Data and code availability

- Raw imaging and electrophysiological data reported in this paper will be shared by the lead contact upon request.
- Original code can be found at https://github.com/hasselmonians/Jiang_Hijazi_2025.
- Any additional information required to reanalyze the data reported in this paper is available from the lead contact upon request.

## STAR★METHODS

### EXPERIMENTAL MODEL AND SUBJECT DETAILS

A total of 53 adult (29 female) C57Bl6j (wild type, WT) mice (obtained from Charles River Ltd, UK) and 14 C1ql2-Cre mice (9 female; heterozygous C57BL/6-C1ql2^em1(cre)Gfng^/J, strain #036955, The Jackson Laboratory) were injected with adeno-associated viruses (AAVs) targeted to the anterior thalamus. Inclusion/exclusion criteria were as follows: *n* = 41/53 mice had viral expression in the ADn and were used for histology; *n* = 12/53 mice were included in pilot behavioral tests; *n* = 36/53 WT mice and 12 C1ql2-Cre mice were included in the main behavioral tests (Figures 2 and S2), *n* = 11/36 WT mice were excluded due to a lack of sufficient viral expression in the ADn; *n* = 32/53 WT mice and 4/14 C1ql2-Cre mice were included for *in vivo* recordings to screen for HD cells, *n* = 2/53 WT mice were used for *in vitro* recordings (not reported), *n* = 11/32 WT mice were excluded due to a lack of viral expression in the recording locations; 26 mice that were included in behavioral tests and/or *in vivo* recordings are reported in Table S1. Recordings from excluded mice will be reported elsewhere.

All animal procedures were approved by the local Animal Welfare and Ethical Review Body under approved personal and project licenses in accordance with the Animals (Scientific Procedures) Act, 1986 (UK) and associated regulations. Animals were maintained in individually ventilated cages on a 12/12 h light-dark cycle (lights on at 07:00). They were typically group-housed (4–5 mice per cage) to prevent detrimental effects of social isolation. Individual cages contained mice from both groups with same sex. Room temperature was maintained at 21°C and humidity was between 50% and 60%. Mice were fed on a standard RM3 diet (pellets, product 801700, Special Diets Services).

Human brain samples (*n* = 2 cases) were obtained from the MRC London Neurodegenerative Diseases Brain Bank (King’s College London, UK) under ethics licenses 18/WA/0206 and 23/WA/0124. The two cases correspond to Case 1 (Braak stage 0, 86 year old male) and Case 16 (Braak stage V, 71 year old male) reported by Sarkany et al.^13^

### METHOD DETAILS

#### Surgical procedures

Mice (5–9 weeks old) were anesthetized with isoflurane, the scalp was clipped, and a subcutaneous injection of Buprenorphine (0.1 mg/kg dose, Vetergesic, Ceva) was administered. Mice were fixed to a stereotaxic frame via ear bars and a jaw bar. Anesthesia was maintained with 1–3% (v/v) isoflurane and body temperature was regulated with a homeothermic blanket (Harvard Apparatus). Ocular lubricant was applied to the eyes, and Bupivacaine (Marcaine, Aspen) was injected into the scalp. An incision was made along the scalp to expose the skull. Craniotomies were made using a surgical microdrill at the following coordinates to target the ADn bilaterally (mm from bregma): −0.82 antero-posterior (AP), ±0.75 medio-lateral (ML). A glass pipette was gradually lowered to 2.8 mm below the brain surface and 100–200 nL of AAV was pressure injected; the pipette was withdrawn 5 min after injection. For Cohort 1, either Tau AAV (AAV8-CBh>EGFP:T2A:Tau(P301L):WPRE, 1.335 × 10^13^ GC/mL) or control GFP AAV (AAV8-CBh>EGFP:WPRE, 5.9 × 10^12^ GC/mL) were injected. For Cohort 2, C1ql2-Cre mice were injected either with a mixture (1:1) of Tau AAV (AAV8-hEF1α-DIO-hMAPT(P301L)-WPRE, 6.9 × 10^12^ GC/mL) and GFP AAV (AAV1-hSyn1-DIO-EGFP-WPRE, 6.7 × 10^12^ GC/mL). After the injections, the scalp was sutured and animals recovered in a cage over a heated blanket.

#### Behavioral tests

All behavioral tests were carried out during the daytime. Mice were first handled for 3 days before initiating behavioral testing and were subjected to a battery of behavioral tests following the order in Figure S2A: open field test (Day 1), Y maze (Day 2), T-maze (Days 8–23, following 3 days of water restriction and 2 days of habituation) and MWM (Days 24–34, 48). All tests except for the T-maze and MWM were performed as previously described.^105^ Behavioral tests were recorded using a camera (Microsoft LifeCam, 30 frames/s), and parameters were extracted and analyzed using AnimalTA software.^108^

##### Open field test

Mice were placed individually in the center of an open field chamber (consisting of an open 50 × 50 cm white square arena and 30 cm high black plastic walls) for 5 min. The room was illuminated using lamps in the ceiling above the arena (340–460 lux light intensity). The arena was thoroughly cleaned with scentless Anistel between subjects. The center area was defined as the 25 × 25 cm interior portion of the square arena. Each mouse was placed gently in one corner of the arena. The total distance traveled (pathlength, in cm) and time spent in the center area were quantified.

##### Spatial novelty preference Y-maze

The task was designed to test spontaneous exploration and the innate preference for exploring a novel environment, as previously reported.^54,105^ A solid black plastic Y-maze (consisting of three 30 cm × 7 cm arms with 20 cm high walls, 160–320 lux light intensity) was positioned 17 cm above the floor. The maze had no intra-maze cues, except for a 20 cm high magenta block that obstructed one of the arms during the sample phase. Prominent extra-maze cues were present.

The test was divided into an exposure phase and a test phase. During the exposure phase, the novel arm was blocked for 5 min, and the mice were placed at the end of the start arm, allowing them to explore the start and other arms. In the test phase, the block was removed when the mice were facing away from it, and they were then free to explore all three arms for an additional 5 min. To prevent the influence of odor cues, the maze was cleaned with Anistel after each mouse’s visit. Time spent and distance traveled (path length) in each arm were analyzed. An entry into an arm was registered when the center of the mouse’s body entered the defined arm area.

##### Non-matching-to-place T-maze

Spatial working memory was assessed as previously reported.^42,105^ Mice were water-restricted until they reached 85–90% of their initial body weight (around 3 days). During this time, mice were habituated to the water reward, which would subsequently be used as a reward in the non-matching-to-place T-maze task. Before the training phase, mice were habituated for 10 min to the T-maze apparatus (50 × 10 cm start arm and two identical 30 × 10 cm goal arms; walls were 10 cm high, and the maze was raised 44 cm off the floor, with a light intensity of 330–520 lux). The rewards consisted of 0.5 mL of water in small plastic dishes, placed at the ends of both goal arms. The habituation was done first in groups (with cagemates) and then individually.

The habituation stage was followed the next day by the training stage, which consisted of 4 trials per day (defined as a session) with each trial having two separate phases (sample and choice phases). In the sample phase, the presence of a block forced the mouse to visit one arm where it was free to retrieve its reward (0.1 mL of water). After reward consumption, mice were returned to their home cage for around 15 s and the T-maze was quickly cleaned. This was followed by the choice phase in which the block was removed and the mouse was free to visit both arms: either visiting the previously unvisited arm to obtain a second 0.1 mL reward or revisiting the previous arm, which was now unrewarded. A pseudorandom sequence was used to assign the rewarded arm, with the same numbers of left or right turns per session.

##### Morris water maze

Following two days of recovery from the water restriction protocol, mice were trained on the classical MWM protocol. Mice were placed facing the sidewall of a plastic pool (120 cm diameter, 30 cm high walls, 30–100 lux light intensity) filled with water (15 cm depth, ~22°C) and containing a hidden platform (10 cm diameter, 14 cm high) submerged in the water. Each training day consisted of four trials during which mice were placed at four different locations of the pool (N, W, E, or S) as the start points. The order of the start points was randomized each day for both groups. The hidden platform was always located in the quadrant between N and W (defined as the target quadrant, TQ). Mice were given 60 s to find the hidden platform. In the event of an unsuccessful search, mice were gently guided to the hidden platform where they remained for around 10 s. The next trial was then initiated. Mice were placed back in their home cage after 2 trials for an inter-trial period of ~2 min before completing trials 3 and 4. Mice were thoroughly dried, and cages were continuously placed on a heat-pad to keep the mice warm between trials. The escape latency during training was assessed as the average time spent to find the hidden platform across the four trials. All trials were recorded via a video camera, and the swim path was quantified using AnimalTA. All experimenters were blind to the experimental group.

Using the tracking data extracted via AnimalTA,^108^ we conducted a detailed analysis of swim trajectories during training. Meander score is the estimation of the path deviation or curvature in the swimming path of the animal during training. This value, automatically extracted using AnimalTA, is the average of the change in direction (turning angle) divided by the distance traveled (path length). The turning angle is the average of angular deviations over the entire pathlength. Exploration represents the total number of pixels the mouse occupied in the MWM (i.e., coverage of the mouse), with each visited pixel counted once. Loops, defined as a mouse swimming in a small circle along a swim path, were manually counted for every trial.

On the 5th day, the hidden platform was removed and mice were released at the opposite quadrant to the normal platform location, facing the wall for the probe test (P1). The trial lasted for 60 s and the time spent and distance swam in each quadrant of the pool were quantified. Other parameters that were extracted were as follows: latency to reach the TQ, the number of entries to the TQ. The swim speed and total distance traveled were also quantified.

Next, reversal training was carried out, which consisted of 3 days of training with 4 trials per day. The hidden platform was placed in the opposite quadrant (new TQ) from the original location. After 3 days of reversal training, the hidden platform was removed and another probe test was performed (P2). The same parameters obtained for the classical MWM were extracted for the reversal MWM. Following the reversal probe test, mice from both groups were disoriented by being placed in a small dark box and rotated 16 times in alternating back-and-forth directions at a speed of 180°–270°/s continuously for 60 s in the same room that contained the MWM.^57^ Mice were then immediately placed in the pool for another probe test (disorientation probe test’, P3). Finally, three weeks after P3, mice underwent a final probe test (P4) to examine long-term memory.

#### *In vivo* electrophysiology

##### Habituation

After completing all behavioral tests, mice were handled for 4 days prior to headplate implant surgeries and were habituated to the recording room by being placed on the running disc setup and being free to explore the apparatus at least 5 min per day (Figure 3A).

##### Headplate implant and craniotomies

Mice were prepared as above for the *viral tracing* surgery. After exposing the skull, two M1 screws (Precision Technology Supplies) were fixed into the skull above the cerebellum as anchor points, with one or both used as electrical reference. Another screw was fixed ~1.50 mm anterior of bregma. Screws were sealed with Refobacin bone cement (Zimmer Biomet), and a machined glass-reinforced plastic D-shaped headplate (custom made at the Department of Physics, Oxford University) was positioned over the screws and secured with bone cement. Mice were administered a subcutaneous injection of 0.5 mL 5% (w/v) glucose in saline (Aqupharm 3, Animalcare) peri-operatively. Craniotomies were made using a surgical microdrill at the following coordinates (mm from bregma): Hippocampal CA1, −2.5 AP, ±1.5 ML; ADn −0.85 AP, ±0.75 ML. The dura was removed with a bent 27-gauge needle. Silicon (Smooth-On) was applied to protect the craniotomy sites by covering the skull inside the headplate, and mice recovered in a cage over a heated blanket. Electrophysiology experiments were initiated the following day.

##### Extracellular recordings with glass electrodes

Mice were head-restrained via a custom-made stainless-steel block (Department of Physics, Oxford University) secured to a heavy-duty frame (model 1430, Kopf Instruments). Head-restrained mice could spontaneously run and rest on a 30 cm diameter plastic running disc covered with paper roll (Figure 3A). The apparatus was mounted to a turntable consisting of a circular aluminum bread-board (Thorlabs Inc) enabling 360° passive rotation. Recordings were performed under photopic conditions (600–700 Lux). Two glass electrodes filled with 3% neurobiotin (w/v) in 0.5 M NaCl (8–18 MΩ) were lowered via the craniotomy sites using IVM-1000 micromanipulators (Scientifica Ltd) to reach the hippocampus and ADn. Signals were amplified ×1000 (ELC-01MX and DPA-2FS modules; npi Electronic GmbH, Tamm, Germany). Wide-band (0.3 Hz–8 kHz) and band-pass filtered (action potentials, 0.8–8 kHz) signals were acquired and digitized at 20 kHz (Power1401; Cambridge Electronic Design Ltd, Cambridge, UK). HumBugs (Digitimer Ltd) were used to remove 50 Hz noise. Speed was measured using a rotary encoder (HEDM-5500#B13, Avago Technologies) underneath the running disc. An inertial measurement unit (SparkFun OpenLog Artemis, which is preprogrammed to automatically log data from Global Navigation Satellite System navigation data) attached to the turntable enabled monitoring HD data at 10 Hz. Electrophysiology data were recorded using Spike2 software (Cambridge Electronic Design). Local field potentials (LFPs) were recorded from the stratum pyramidale of the CA1 region of the hippocampus, identified by the combination of positive sharp-waves and 130–230 Hz ripples during rest periods and 5–12 Hz theta oscillations during movement periods.^105^ The ADn was targeted bilaterally in each mouse. To localize the ADn, the glass electrode was lowered through the craniotomy sites to the target depth (~2.5 mm). HD cells were detected by slowly rotating the apparatus until an abrupt increase in firing was observed when the mouse faced a specific direction. The firing activity was then recorded while the mouse was manually rotated both clockwise and counterclockwise to acquire firing data across all angles. Cells of interest were then juxtacellularly labeled with 200 ms positive current pulses, followed by a recovery period of 2–6 h.

##### Acute silicon probe recordings

Following glass electrode recordings and localization of the ADn, methylene blue was applied to the tip of the glass electrode and lowered to the same AP-ML position in the craniotomy site to ensure proper localization of the probe. Then an acute 16-channel silicon probe (A1×16-poly 2s probe, NeuroNexus) connected to an RA16-AC preamplifier (Tucker-Davis Technologies) was carefully painted with DiI and positioned at the exact location marked by the methylene blue tracer then lowered to the same depth as the previous glass electrode. Signals were amplified ×1000 (Lynx-8 amplifiers, Neuralynx), band-pass filtered (0.5 Hz–8 kHz), digitized at 20 kHz with a Power1401 and recorded with Spike2. Silicon probe recording sites were confirmed by examining DiI fluorescence in serially processed brain sections.

#### Histology

##### Transcardial perfusion and sectioning

Mice were deeply anesthetized with sodium pentobarbital (50 mg/kg, i.p.) and transcardially perfused first with saline followed then with 4% paraformaldehyde, 15% v/v saturated picric acid, and 0.05% glutaraldehyde in 0.1 M phosphate buffer (PB), pH 7.4. Brains were removed from the skull. Some brains were post-fixed overnight in fixative lacking the glutaraldehyde. After washing in 0.1 M PB, 70 μm coronal sections were cut using a Leica Microsystems VT 1000S vibrating blade microtome and stored in 0.1 M PB with 0.05% sodium azide at 4°C.

##### Streptavidin visualization and immunohistochemistry

Using the indirect primary antibody detection method in combination with fluorochrome-conjugated secondary antibodies, the expression of cell-type specific molecules was tested on individually labeled neurons and on control tissue.

For the visualization of neurobiotin-labeled cell processes, brain sections were permeabilized in Tris-buffered saline (0.9% NaCl buffered with 50 mM Tris, pH 7.4; TBS) with 0.3% Triton X-100 (TBS-Tx) or via rapid 2× freeze–thaw (FT) over liquid nitrogen (cry-oprotected in 20% sucrose in 0.1 M PB) then streptavidin-conjugated Cy3 or Cy5 was applied at 1:500 dilution in TBS-Tx (or TBS if permeabilized with FT) for 4 h at room temperature (RT) or overnight at 4°C. Sections were washed in TBS/TBS-Tx and mounted to glass slides in Vectashield (Vector Laboratories).

First, sections were blocked for 1 h in 20% normal horse serum (NHS) in TBS/TBS-Tx followed by 2–6 days incubation at 4°C in primary antibody solution containing 1% NHS in TBS/TBS-Tx. The following primary antibodies (and dilutions) were used: rabbit anti-T22 1:1000 (ABN454, Merck), mouse anti-HT7 1:1000 (MN-1000, Pierce Thermo Scientific), rabbit anti-4R tau 1:1000 (ab242333, abcam), rabbit anti-Tau (phospho T231) 1:1000 (ab151559, Abcam), rabbit anti-C1ql2 1:1000 (NBP2–34090, Novus Biologicals), guinea pig anti-VGLUT2 1:1000 (135 404, Synaptic Systems), guinea pig anti-Parvalbumin 1:1000 (195004/1–19, Synaptic Systems). Subsequently, sections were washed 3 times in 0.1 M PB, then incubated in secondary antibody solution containing 1% NHS in TBS/TBS-Tx for 4 h RT or overnight at 4°C. The following secondary antibodies (and dilutions) were used in various combinations (all raised in donkey): anti-mouse and anti-rabbit Alexa Fluor 405 1:250 from Invitrogen, anti-guinea pig DyLight 405 (706–475-148), anti-guinea pig, anti-rabbit, and anti-mouse Alexa Fluor 647 1:500 (706–475-148, 711–605-152, 705–605-151) from Jackson ImmunoResearch. After 3 washes in 0.1 M PB, sections were mounted in Vectashield.

For diaminobenzidine (DAB)-based horseradish peroxidase (HRP) reactions, sections were blocked for 1 h in 20% normal goat serum (NGS) in TBS followed by incubation with primary antibodies in TBS containing 1% NGS for 2 d at 4°C. After incubation with primary antibodies, sections were rinsed three times in TBS and blocked for 10 min at RT in 1% H_2_O_2_ solution in 0.1 M PB to reduce non-specific background reactions. Next, sections were incubated overnight at 4°C in 1:100 biotinylated goat anti-rabbit secondary antibody (Vector Labs, BA-1000) in TBS containing 1% NGS. After repeated washes in TBS, sections were incubated for 3 days at 4°C in Avidin+Biotin-HRP complex (Vectastain ABC Elite kit, Vector Laboratories) in TBS. Subsequently, peroxidase was visualized using a mix of 1% nickel ammonium sulfate, 0.4% ammonium-chloride, and 3,3-DAB (0.5 mg/mL, Sigma-Aldrich) developed with 0.01% H_2_O_2_. After washing sections in PB, sections were treated with 0.25% OsO_4_ in 0.1 M PB for 5 min. Next, after washing in 0.1 M PB at least four times, some sections were transferred onto slides in chrome alum gelatin and dried in air. To avoid air bubbles, sections were then incubated in fresh xylene for 10 min before being quickly mounted in DePeX mounting medium (Merck). Other sections were dehydrated using an ascending ethanol series followed by acetonitrile. This was followed by embedding in epoxy resin (Durpucan AMC, Fluka, Sigma-Aldrich), incubating overnight at RT then transferring onto slides. For polymerization, sections were incubated at 60°C for 2 d.

##### Formalin-fixed paraffin-embedded (FFPE) sections

Slide-mounted brain sections (5 μm thick) were obtained from FFPE tissue blocks using a microtome (Reichert-Jung, 2035). Sections were deparaffinized in 100% xylene and rehydrated in a descending ethanol series (100%, 95%, 70%, 50%), followed by antigen retrieval (10 mM Sodium Citrate Buffer at pH 6 at 90°C for 30 min).

For immunofluorescence, sections were blocked in 20% NHS, followed by a 3-day incubation in primary antibody solution in 0.1 PB at 4°C. Sections were washed 3 times in 0.1 M PB then incubated in secondary antibody solution in 0.1 M PB for 1 h at room temperature (RT) or overnight at 4°C. Finally, sections were mounted in Vectashield.

For brightfield immunohistochemistry, sections were blocked in 4% NGS in 0.1M PB for 1 h, followed by incubation in primary antibody solution for 1–3 days. After washing in 0.1 M PB, sections were incubated with a biotinylated secondary antibody in 0.1 M PB overnight at 4°C. Next, sections were incubated with 1:100 Avidin+Biotin-HRP complex in 0.1M PB at 4°C then processed using 0.5 mg/mL DAB, 2% nickel ammonium sulfate, and 0.4% ammonium chloride in 0.1 M PB. Hydrogen peroxide was added to a final concentration of 0.002% w/v. After ~15 min, reactions were stopped by washing 3 × 10 min in 0.1 M PB. Finally, sections were incubated in xylene for 5 min and mounted in DePeX.

#### Microscopy

##### Widefield epifluorescence and confocal laser scanning microscopy

The immunohistochemical reactions were first evaluated using widefield epifluorescence on a Leitz DMRB microscope (Leica Microsystems) equipped with PL Fluotar objectives (magnification/numerical aperture: 5×/0.15, 10×/0.3, 20×/0.5, 40×/0.7; OpenLab software) or an AXIO Observer Z1 microscope (LSM 710; Zeiss) equipped with Plan-Apochromat 10×/0.3, 20×/0.8 and 40×/1.4 objectives (Axiovision or ZEN Blue 2.6 software). To improve image resolution and increase contrast, when needed, reactions were further assessed using confocal laser scanning microscopy; the LSM 710 was used with Plan-Apochromat 40×/1.4, 63×/1.4, and 100×/1.46 objectives (ZEN Black 14.0 software). Laser lines (solid-state 405 nm, argon 488 nm, HeNe 543 nm and HeNe 633nm) were configured with the appropriate beamsplitters. The pinhole was set to ~1 Airy Unit for each channel.

### QUANTIFICATION AND STATISTICAL ANALYSIS

#### Classification of HD cells

Neurons that exhibited bursting non-rhythmic firing that was clearly related to the animal’s head direction were initially selected for recording, which biased the initial search strategy to HD cells of the ADn. By visualizing labeled cells, we confirmed that recorded cells from either the same penetration site or from closely aligned coordinates were located in the ADn. For glass electrode single-cell data, spikes were isolated by thresholding high-pass filtered voltage traces of their peaks and validated by using principal component analysis and/or visual inspection in Spike2 software (Cambridge Electronic Design, Cambridge, UK).

Analysis procedures were applied to both glass electrode single-cell data and silicon-probe data. Inclusions criteria: we analyzed recordings that were longer than 100 s, were from recording sites in the ADn that had GFP expression (Table S1), and had head direction coverage of at least one full turn with all angles represented. The directional tuning curve for each cell was obtained by plotting the firing rate as a function of the mouse’s directional heading, divided into bins of 6°. The firing rate was computed based on the total number of spikes divided by the total time in that bin. All HD properties-related parameters were analyzed based on published parameters^16^ using customized Python scripts. The mean vector length (*r*) is a measure of the non-uniformity (or directionality) of the directional tuning curve and can vary between 0 (a uniform distribution) and 1 (a non-uniform distribution). Mean vector lengths were used to determine the extent of uniformity in the distribution of firing rate bins. Based on the published criteria,^55,66^ subjective assessment of the distribution of *r*, and each cell’s directional tuning curves, a criterion of *r* ≥ 0.3 and peak firing rate >1 Hz was used to classify HD cells. For some cells and units that only showed directionality within a specific rotation direction (either *r*_CW_ (clockwise) or *r*_CCW_ (counterclockwise) > 0.3 and |*r*_CW_- *r*_CCW_| > 0.2), only spikes or units recorded during the most directionally responsive rotation direction were analyzed. Further details of HD cells exhibiting direction selectivity will be reported elsewhere.

The directional tuning curve was computed and fitted with von Mises distributions.^66,109^ Five parameters were analyzed: (1) the preferred firing direction – the head direction angle with the highest firing rate; (2) peak firing rate – the highest firing rate from the directional tuning curve, which indicates the firing rate when the mouse is facing in the cell’s preferred direction; (3) directional tuning width – the range of head directions over which the cell fires; (4) background firing rate – the average firing rate when the mouse is facing outside of the directional firing range of the cell; (5) directional coherence – the smoothness of the raw directional tuning curve.^24^ All polar plots presented in this paper are based on tuning curves smoothed with a Gaussian filter (σ = 1.5).

Another plot showing the number of spikes versus time was constructed to compute two parameters: (1) the maximum firing rate, which is defined as the highest firing rate within a 200 ms time bin from the entire recording session; (2) the mean firing rate, which is the average firing rate of the cell over the entire recording session.

#### Burst analysis

Burst analysis was performed using a burst analysis script in Spike2 (https://ced.co.uk/downloads/scriptspkanal). For each HD cell, spikes were extracted from time periods where the mouse was resting and facing the preferred firing direction. The minimum duration of the recording was 20 s, and the minimum detected bursts were set to 4. Recordings that did not meet these thresholds were excluded. The minimum number of spikes per burst was set to 3. The maximum interval between two spikes that signifies the start of a burst was set to 50 ms. The longest interval between two spikes (ISIs) *within* a burst (i.e., intervals longer than this terminate a burst) was set to 30 ms.

The following parameters were extracted: percentage of spikes per burst (number of spikes within bursts/number of all spikes); the mean number of spikes per burst (average number of spikes within bursts); the mean burst length (average duration of bursts); the inter-spike interval during burst (duration between spikes during a burst); the inter-burst duration (duration between two consecutive bursts); and the mean cycle period (average period between bursts, cycle period = inter-burst duration + burst length).

The burst index and CV were analyzed using customized Python scripts. To compute the burst index, the number of ISIs that were <30 ms, but >2 ms due to the refractory period was counted, and then this value was divided by the total number of ISIs that occurred between 0 and 100 ms.^24^ The CV was calculated by dividing the standard deviation of all the ISIs by the mean ISI.

#### Silicon probe data analysis

Similar to the analysis of glass electrode recorded cells, only recordings from a DiI track confirmed to be within the ADn along with GFP expression and whose head direction coverage included at least one full turn were included in the analysis (Table S1). Spikes were detected, sorted, and clustered offline using Kilosort2,^107^ in MATLAB. Subsequently, interactive visualization and manual curation of the data were carried out using Phy2 based on refractory periods, spike waveform and cross-correlations. In order to obtain well-isolated and stable units, only units with mean firing rates >1 Hz, refractory-period contamination <2 ms, and consistent spike waveforms but dissimilar to nearby clusters were included in the analysis.

#### LFP analysis

LFP recordings with a regular synchronized theta rhythm and non-theta epochs containing sharp wave ripples were selected for LFP analysis. For LFP channels, direct current shifts were removed with 0.1 s sliding windows then channels were downsampled to 1.25 kHz in Spike2 and were exported for analysis using custom scripts in Python and Mathematica. For each animal, 1 to 5 theta epochs were sampled per recording, ranging from 8 to 109 s to define each epoch. We excluded epochs that had 50 Hz electrical noise. Empirical mode decomposition (EMD) was carried out in Python using the emd package (https://gitlab.com/emddev/emd).^110^ To obtain intrinsic mode functions (IMFs), the following masks were used for masked EMD (emd.sift.mask_sift): 350, 200, 70, 40, 30, 7, 1, divided by the sampling rate. Instantaneous amplitude, frequency and phase were obtained for each IMF using emd.spectra.frequency_transform with the Normalized-Hilbert Transform (nht). For IMF6, values obtained per mouse were binned and averaged for low running speeds (1–4 cm/s) and ‘high’ speeds (4–10 cm/s).

#### Quantification (histology)

A semi-quantitative scoring of viral GFP expression (Table S1) was performed using the following criteria: (−), no GFP positive cells in the ADn; (+), small number of GFP positive cells in the ADn or projections; (++), moderate number of GFP positive cells in the ADn; (+++), large number of GFP positive cells. This was done for each hemisphere and the score per mouse was an average of both hemispheres. Colocalization of C1ql2 and NeuN in the ADn was quantified using the Cell Counter plugin in ImageJ based on 5 sections per mouse from *n* = 3 mice. Colocalization of GFP and T22 was based on 2 sections per mouse from *n* = 4 ADn-gfp mice and *n* = 4 And-tau mice from Cohort 1, and *n* = 2 ADn-gfp mice and *n* = 6 ADn-tau mice from Cohort 2. Coverage of GFP was calculated by dividing the total number of GFP+ cells by the total number of C1ql2+ cells, based on 3 sections (confocal z stack) from *n* = 2 ADn-gfp mice and *n* = 4 ADn-tau mice from Cohort 2 (representing the anterior, medial and posterior regions of the ADn). Coverage of T22 was calculated by dividing the total number of T22+ cells by the total number of NeuN+ cells, based on 2–3 sections from *n* = 2 ADn-gfp mice and *n* = 4 ADn-tau mice from Cohort 2.

#### Statistics

All data are represented as mean ± SEM or median [IQR]. Experimental units (e.g., mice, cells) are specified in the text after the *n* values. Statistical analysis was carried out in GraphPad Prism and Python. The alpha was set to 0.05. For data that approximated a normal distribution (tested by the Shapiro-Wilk test), unpaired Student’s t tests were used to compare two groups with equal variances and unpaired t tests with Welch’s correction were used in two groups having different variances, otherwise Mann-Whitney *U* tests were used. For comparisons of more than two groups, we used Analysis of Variance (ANOVA) for parametric data followed by Fisher’s LSD *post-hoc* test. Spearman’s correlation was used to test the correlation between two ranked variables.

## Supplementary Material

1

2

3

5

6

7

8

9

10

11

12

SUPPLEMENTAL INFORMATION

Supplemental information can be found online at https://doi.org/10.1016/j.celrep.2025.116610.

## Figures and Tables

**KEY RESOURCES TABLE:** 

REAGENT or RESOURCE	SOURCE	IDENTIFIER

Antibodies		

Rabbit polyclonal anti-T22	Merck	ABN454; RRID: AB_2888681
Mouse monoclonal anti-HT7	Pierce Thermo Scientific	MN-1000; RRID: AB_2314654
Rabbit monoclonal anti-4R tau	Abcam	ab218314; RRID: AB_3068614
Rabbit monoclonal anti-P-tau (phospho-tau T231)	Abcam	ab151559; RRID: AB_2893278
Mouse monoclonal anti-AT8 (phospho-tau Ser202 Thr205)	Invitrogen	MN1020; RRID: AB_223647
Guinea pig polyclonal anti-VGLUT2	Synaptic Systems	135 404; RRID: AB_887884
Guinea pig polyclonal anti-parvalbumin	Synaptic Systems	195 004; RRID: AB_2156476
Rabbit polyclonal anti-C1ql2	Novus Biologicals	NBP2-34090; RRID: AB_3285559

Chemicals, peptides, and recombinant proteins		

Ultra-purified recombinant AAV8 virus, pilot-scale packaging pAAV-CBh>EGFP:WPRE	Vector Builder	AAV8SP(VB900088-2238xse)-C Lot 211102AAVJ09 https://en.vectorbuilder.com/vector/VB900088-2238xse.html
Ultra-purified recombinant AAV8 virus, pilot-scale packaging pAAV-CBh>EGFP:T2A:Tau(P301L):WPRE	Vector Builder	AAV8SP(VB211015-1033pqq)-C Lot 211117AAVD11 https://en.vectorbuilder.com/vector/VB211015-1033pqq.html
AAV-8/2-hEF1α-dlox-dTomato-EGFP(rev)-dlox-WPRE-hGHp(A)	VVF Zurich	Custom made from the plasmid
AAV-8/2-hEF1α-dlox-hMAPT(P301L)(rev)-dlox-WPRE-hGHp(A)	VVF Zurich	Custom made from the plasmid (Addgene: pAAV-FLEX-P301L Tau)
Neurobiotin tracer	Vector Laboratories	SP-1120; RRID: AB_2313575
Isoflurane inhalation anesthesia	Teva Ltd.	https://products.tevauk.com/p/Category?id=185
Vetergesic (buprenorphine)	Ceva	56454-04
Marcain (bupivacaine)	Aspen	PS22421
Metacam (meloxicam)	Boerhinger Ingelheim	N/A
Refobacin bone cement	Zimmer Biomet	3003940002-3
Tetric EvoFlow dental cement	Ivoclar Vivadent	A1
DiI tracer	Life Technologies	V22889
Normal horse serum	Vector Laboratories	S-2000
Normal goat serum	Vector Laboratories	S-1000

Critical commercial assays		

Vectastain ABC Elite kit	Vector Laboratories	PK6100; RRID: AB_2336819
Vectashield Antifade Mounting Medium	Vector Laboratories	H-1000; RRID: AB_2336789

Experimental models: Organisms/strains		

C57BL6j mice	Charles River Laboratories	https://www.criver.com/
C1QL2-IRES-Cre mice (C57BL/6-C1ql2^em1(cre)Gfng^/J)	Jackson Laboratory	https://www.jax.org/strain/036955

Software and algorithms		

FIJI (ImageJ)	NIH	https://imagej.net/software/fiji/
MATLAB	Mathworks	https://www.mathworks.com/products/matlab.html
Python		https://www.python.org/
Zen Black, Zen Blue, Axiovision	Zeiss	www.zeiss.co.uk
Spike2	Cambridge Electronic Design	http://ced.co.uk/
Kilosort2	Pachitariu et al.^107^	https://github.com/MouseLand/Kilosort
Phy		https://github.com/cortex-lab/phy
GraphPad Prism 7	GraphPad Software	https://www.graphpad.com/
Analysis and plotting codes	This paper	https://github.com/hasselmonians/Jiang_Hijazi_2025
AnimalTA	Chiara and Kim^108^	https://github.com/VioletteChiara/AnimalTA

Other		

A1x16-poly 2s silicon probe	NeuroNexus	https://neuronexus.com/
ASSY-37 P-1 silicon probe	Cambridge Neurotech	https://www.cambridgeneurotech.com/neural-probes/probe-maps
ASSY-37 H-Series 6b silicon probe	Cambridge Neurotech	https://www.cambridgeneurotech.com/neural-probes/probe-maps

## Highlights

- ADn-tau mice are defined by human mutant tau in the anterodorsal thalamic nucleus
- ADn-tau mice exhibit disorientation during spatial memory acquisition
- ADn cells in ADn-tau mice have lower directionality and directional coherence
- Head direction cells in ADn-tau mice have altered burst firing
