# Supplementary material for: Pathological tau alters head direction signaling and induces spatial disorientation
Source: Cell Rep. Author manuscript; Available in PMC 2026 Jun 14. (PMC13265041; doi:10.1016/j.celrep.2025.116610)
Supplement: 1 [file NIHMS2132733-supplement-1.pdf]

Cell Reports, Volume *44*

Supplemental information

Pathological tau alters head direction signaling

and induces spatial disorientation

Shan Jiang, Sara Hijazi, Barbara Sárkány, Verena G. Gautsch, Patrick A. LaChance, Michael E. Hasselmo, David Bannerman, and Tim J. Viney

Supplementary Figures

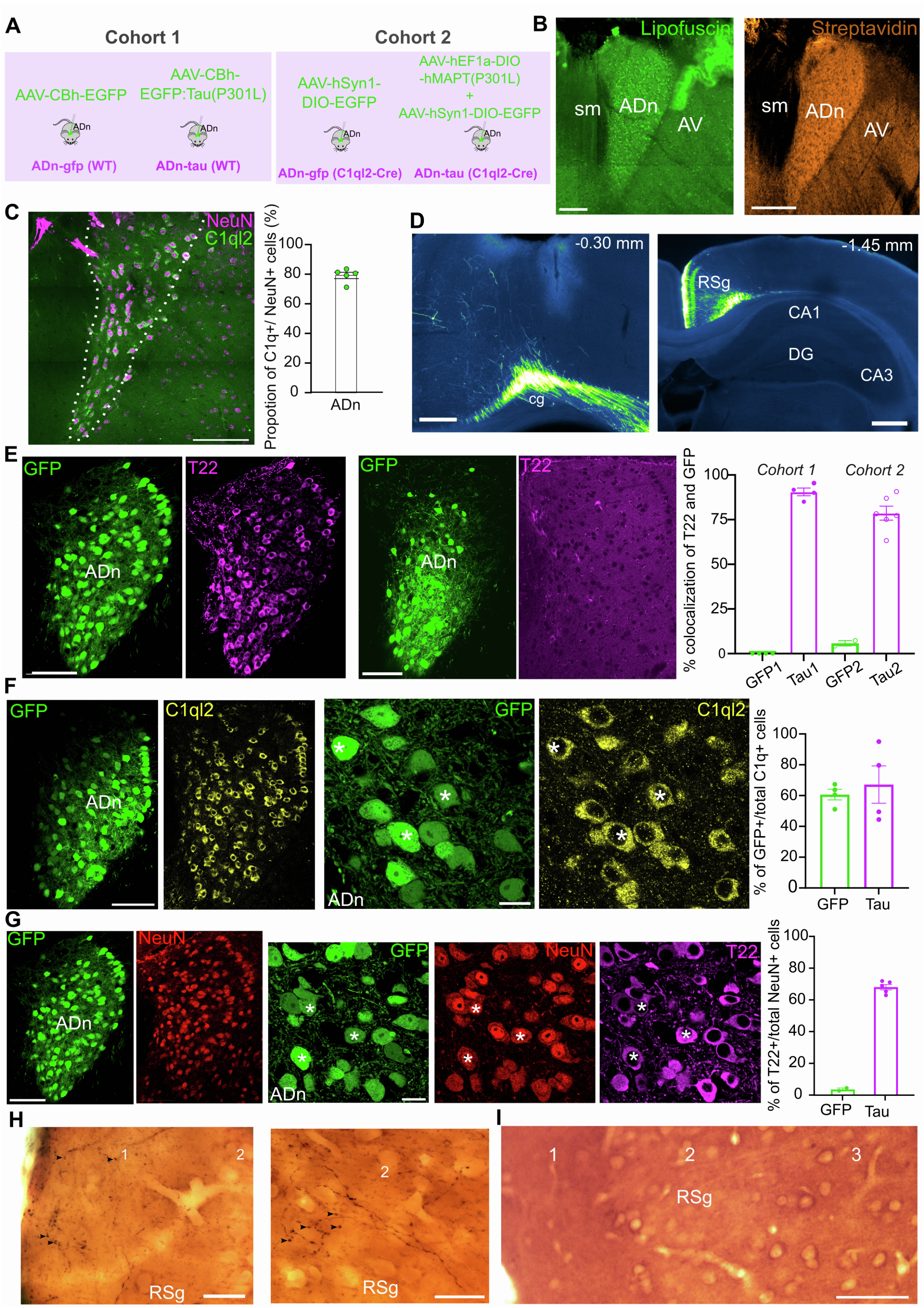

Figure S1. GFP expression and ptau immunoreactivity in ADn-tau and ADn-gfp mice, related to Figure 1.

(**A**) Schematic of experimental design for Cohorts 1 and 2. (**B**) Left, the ADn has a high level of lipofuscin compared to surrounding brain regions (488 nm excitation, widefield autofluorescence, case SJ14). Right, the ADn has a high density of biotin, visualized by streptavidin-Cy5, compared to neighboring regions (widefield epifluorescence, case SJ25). (**C**) Left, coronal section of mouse ADn showing C1ql2 (green) and NeuN (magenta) immunoreactivity. Case SH49. Right, quantification of C1ql2+/NeuN+ cells (counts from 5 sections containing the ADn from three mice). (**D**) Trajectory of GFP-expressing ADn cell axons to postsynaptic target regions. Axons leave the ADn and branch in the TRN at -0.90 mm from Bregma. They travel rostrally through the striatum and cross the corpus callosum to enter the cingulum bundle (-0.30 mm from Bregma). At the level of dorsal hippocampus (-1.45 mm from Bregma, right panels), ADn cell axons innervate the full extent of the RSg, then continue to other areas including PoS. Note lack of GFP in the hippocampus, dentate gyrus (DG) and other areas of isocortex lateral to RSg. Widefield epifluorescence micrographs, case SH30. (**E**) Left panels, confocal image of the ADn from an ADn-tau mouse (Case SH71) showing colocalization of GFP (green) and T22 (magenta). Middle panels, lack of T22 immunoreactivity in the ADn from an ADn-gfp mouse (Case SH73). Maximum intensity z-projections (35 μm thick). Right, quantification of T22+ GFP+ cells (from n=4 ADn-gfp mice and n=4 ADn-tau mice from Cohort 1, and n=2 ADn-gfp mice and n=6 ADn-tau mice from Cohort 2). (**F**) Left, GFP+ cells (green) in the ADn colocalize with C1ql2 (yellow). Confocal single optical section, Case SH73, ADn-gfp mouse. Middle, examples of GFP-C1ql2 double positive cells (e.g. asterisks). Right, quantification of the proportion of GFP+ C1ql2+ cells in Cohort 2 (n=2 ADn-gfp mice [GFP] and n=4 ADn-tau mice [Tau]). (**G**) Left, confocal image showing GFP+ cells (green) expressed in the majority of the ADn along with neuron-specific marker NeuN (red). Case SH73, ADn-gfp mouse. Middle, examples of GFP-T22-NeuN triple positive cells in the ADn of an ADn-tau mouse (e.g. asterisks). Right, quantification of the proportion of T22+ NeuN+ cells (n=2 ADn-gfp mice and n=4 ADn-tau mice from Cohort 2). (**H**) T22-immunoreactive axons and axon terminals (e.g. arrowheads) in superficial RSg from an ADn-tau mouse (Case TV176). Brightfield images (minimum intensity projections), DAB-based HRP reaction. (**I**) Lack of T22 immunoreactivity in the RSg from an ADn-gfp mouse (Case TV177). Brightfield image, DAB-HRP reaction. Scale bars (μm): B left 100, right 200; C 200; D left 250, right 500; E 100; F left 100, middle 20; G left 100, middle 20; H 20; I 50. Data are represented as mean ± SEM (C-G).

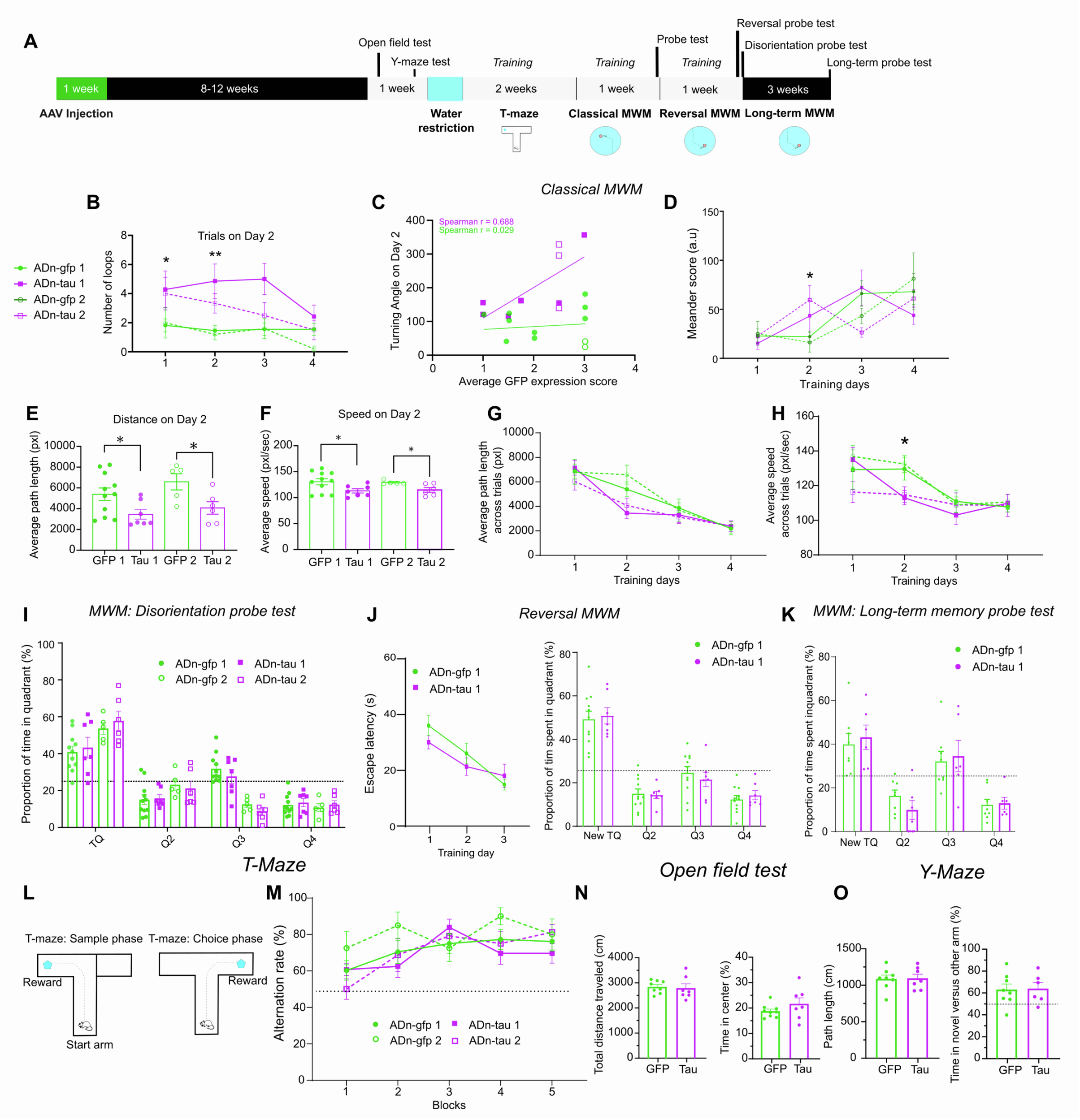

Figure S2. Behavioral tests in ADn-tau and ADn-gfp mice, related to Figure 2.

(**A**) Experimental design. After 8-12 weeks of viral expression, ADn-gfp and ADn-tau mice were tested in a battery of behavioral tasks. (**B**) In the classical MWM, the average number of loops per trial on Day 2 was increased in ADn-tau mice on the first 2 trials (*group effect* two-way repeated measures ANOVA: $F_{3,30} = 8.474$, ***p <0.001; *post-hoc* LSD test on trial 1 and trial 2). (**C**) Correlation between average GFP expression level and turning angles on Day 2 of training (Spearman r: ADn-gfp, p = 0.929; ADn-tau, *p = 0.035). (**D**) The average meander score was significantly different across groups as an interaction of training days (*group x training effect* two-way repeated measures ANOVA: $F_{1,27} = 4.121$, *$p < 0.05$, *post-hoc* LSD test for Day 2: *p<0.05). (**E**) The average path length to reach the hidden platform was significantly different between groups on Day 2. (**F**) The average speed was also different on Day 2 of training. (**G**) Both groups showed significant learning in terms of average path length to the platform across trials per training day (*training effect* two-way repeated measures ANOVA: $F_{3,30} = 38.2$, ****p <0.0001). (**H**) Average swimming speed per trial of four training

days. (**I**) Proportion of time spent in each quadrant in the disorientation probe test (*quadrant x group interaction effect*: $F_{3,36}$ = 0.364, p = 0.778). (**I**) There was no difference observed in the time spent in the new TQ in the probe test after disorientation. Dashed line, chance level. (**J**) Both groups performed similarly during training in the reversal phase of the MWM (left, *training effect* two-way repeated measures ANOVA: $F_{2,32}$ = 14.97, ***p < 0.0001, *training x group interaction effect*: $F_{2,32}$ = 1.765, p = 0.187) and in the percentage of time spent in the TQ during the reversal probe test (right, ADn-gfp vs chance: U = 6, **p = 0.0040; ADn-tau vs chance: U = 6, *p = 0.0239, ADn-gfp vs ADn-tau: $t_{(16)}$ = 0.4040, p = 0.6916). (**K**) A long-term memory probe test was performed 3 weeks after the disorientation probe test. No differences were observed between groups. Both groups showed a clear preference for the TQ (ADn-gfp vs chance: ***p = 0.0003; ADn-tau vs chance: ***p = 0.0006, ADn-gfp vs ADn-tau: $t_{(16)}$ = 0.4381, p = 0.6691). (**L**) Schematic of the non-matching-to-place (rewarded alternation) T-maze task. Training consisted of 5 sessions (defined as a block of 8 trials over 2 days) conducted over 10 days. Each trial included 1 sample phase (with one arm blocked and the other containing a water reward), and 1 choice phase (both arms open and only the previously unvisited arm rewarded). (**M**) Both groups learned the T-maze task, as shown by the increased alternation rates over time. (**N**) Open field test. No differences were observed between groups for the total distance traveled ($t_{(12)}$ = 0.5889, p = 0.2913) or time spent in the center ($t_{(12)}$ = 1.01, p = 0.0583). (**O**) Spontaneous spatial novelty preference was tested using a Y-maze that consisted of three arms (start arm, other arm, and novel arm). ADn-gfp mice (n=8) and ADn-tau mice (n=6) showed no difference in the total distance traveled (left, $t_{(12)}$ = 0.5559, p = 0.0626) or in the proportion of time spent in the novel arm compared to the other arm (right, N/N+O, $t_{(12)}$ = 1.592, p = 0.1374). Abbreviations: Y-maze, spontaneous novelty preference Y-maze task; T-maze, non-matching-to-place T-maze task; MWM, Morris water maze. Dashed lines, chance levels. Data are represented as mean ± SEM; *p < 0.05, **p < 0.01.

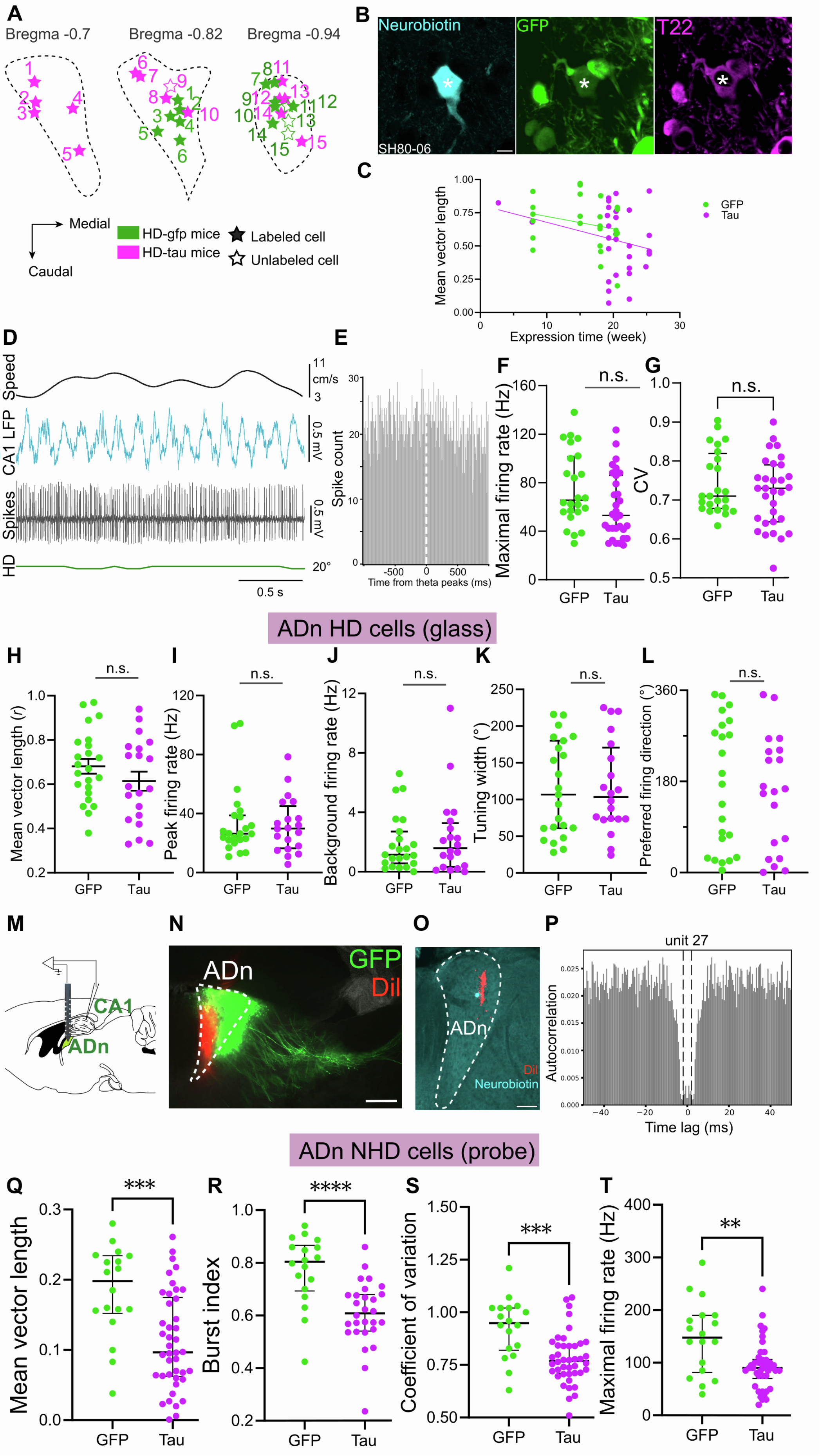

A
Bregma -0.7
Bregma -0.82
Bregma -0.94
Medial
Caudal
HD-gfp mice
HD-tau mice
Labeled cell
Unlabeled cell
B
Neurobiotin
GFP
T22
SH80-06
C
Mean vector length
Expression time (week)
GFP
Tau
D
Speed
cm/s
CA1 LFP
0.5 mV
Spikes
HD
20°
0.5 s
E
Spike count
Time from theta peaks (ms)
F
Maximal firing rate (Hz)
n.s.
G
CV
ADn HD cells (glass)
H
Mean vector length (r)
I
Peak firing rate (Hz)
J
Background firing rate (Hz)
K
Tuning width (°)
L
Preferred firing direction (°)
M
CA1
ADn
N
GFP
DiI
O
Neurobiotin
P
unit 27
Autocorrelation
Time lag (ms)
ADn NHD cells (probe)
Q
Mean vector length
R
Burst index
S
Coefficient of variation
T
Maximal firing rate (Hz)

Figure S3. Recording locations and electrophysiological properties of ADn cells, related to Figure 3.

(**A**) Schematic of the ADn at three representative antero-posterior levels (coronal sections) indicated by the distance from Bregma in mm. Solid stars represent neurobiotin-labeled ADn cells (n=14 from 8 ADn-tau mice, and n=12 from 5 ADn-gfp mice). Unfilled stars represent unlabeled ADn cells recorded near to labeled cells (n=1 from 1 ADn-tau mouse, and n=3 from 1 ADn-gfp mouse). Cell IDs for ADn-tau mice (magenta, from 1-14): TV189-17, TV189-16, TV189-15, SH37-03, SH36-2h, SJ14-10, SH79-07, SH33-2b, SH33-2c, SJ16-10, SH40-061, SH40-08, SH40-071, SH40-062, SH40-05. Cell IDs for ADn-gfp mice (green, from 1-15): TV190-2h, SH46-2d, SH30-10, TV190-2i, SH56-2a, TV191-3f, TV191-3e, SH49-021, SH49-022, SH49-023, SH46-2e, SH56-2c, SH49-031, SH46-2f, SH46-2g. (**B**) Single confocal image of recovery of a juxtacellularly labeled ADn cell (neurobiotin, cyan, cell SH80-06, asterisk) that expressed GFP (green) and was immunoreactive for oligomeric tau (T22, magenta). Scale bar, 20 µm. (**C**) Correlation of mean vector length and viral expression time. (**D**) Spiking activity of an ADn HD cell (SH33-1h1) in the preferred firing direction during locomotion with theta oscillations in CA1. The cell lacked theta-rhythmic firing. (**E**) Cross-correlogram between CA1 theta and ADn HD cell spikes (SH33-1h1), $p < 0.05$. (**F-G**) No significant differences for maximal firing rates and CV between ADn cells recorded from ADn-gfp (green) and ADn-tau (purple) mice with glass electrodes. (**H-L**) Directional properties of ADn HD cells recorded with glass electrodes (**H**, mean vector length; **I**, peak firing rate; **J**, background firing rate; **K**, directional tuning width; **L**, preferred firing direction). Peak firing rate: 2.99 [1.6-4.5] Hz from ADn-tau mice versus 2.63 [2.2-3.8] Hz from ADn-gfp mice, $U = 223$, $p = 0.8708$. Background firing rate: 0.16 [0.03-0.32] Hz from ADn-tau mice versus 0.12 [0.05-0.27] Hz from ADn-gfp mice, $U = 217$, $p = 0.7586$. Preferred firing direction: 411.5 ± 145.4° from ADn-tau mice versus 363.9 ± 85.9° from ADn-gfp mice, $U = 200$, $p = 0.4765$. (**M**) Schematic showing *in vivo* silicon probe recordings in the ADn with a glass electrode in hippocampal CA1. (**N**) Widefield fluorescence micrograph of a coronal brain section showing viral expression of GFP (green) and a silicon probe tract (DiI, red) localized to the ADn, case SH46. Scale bar, 200 µm. (**O**) A coronal brain section showing a neurobiotin-labeled HD cell (SH33-2c, cyan) and a probe tract (DiI, red) localized to the ADn. Scale bar, 100 µm. (**P**) The 100 ms autocorrelogram for all spikes from unit 27 from mouse SH46. Bin size, 0.05 ms; window size, 50 ms; refractory period, 2 ms. (**Q-T**) Properties of NHD cells recorded with silicon probes (**Q**, mean vector length; **R**, burst index; **S**, coefficient of variation; **T**, maximal firing rate). Data are represented as mean ± SEM (H, Q, R, S) or median and IQR (I, J, K, T); $^{**}p < 0.01$, $^{***}p < 0.001$, $^{****}p < 0.001$, n.s., not significant.

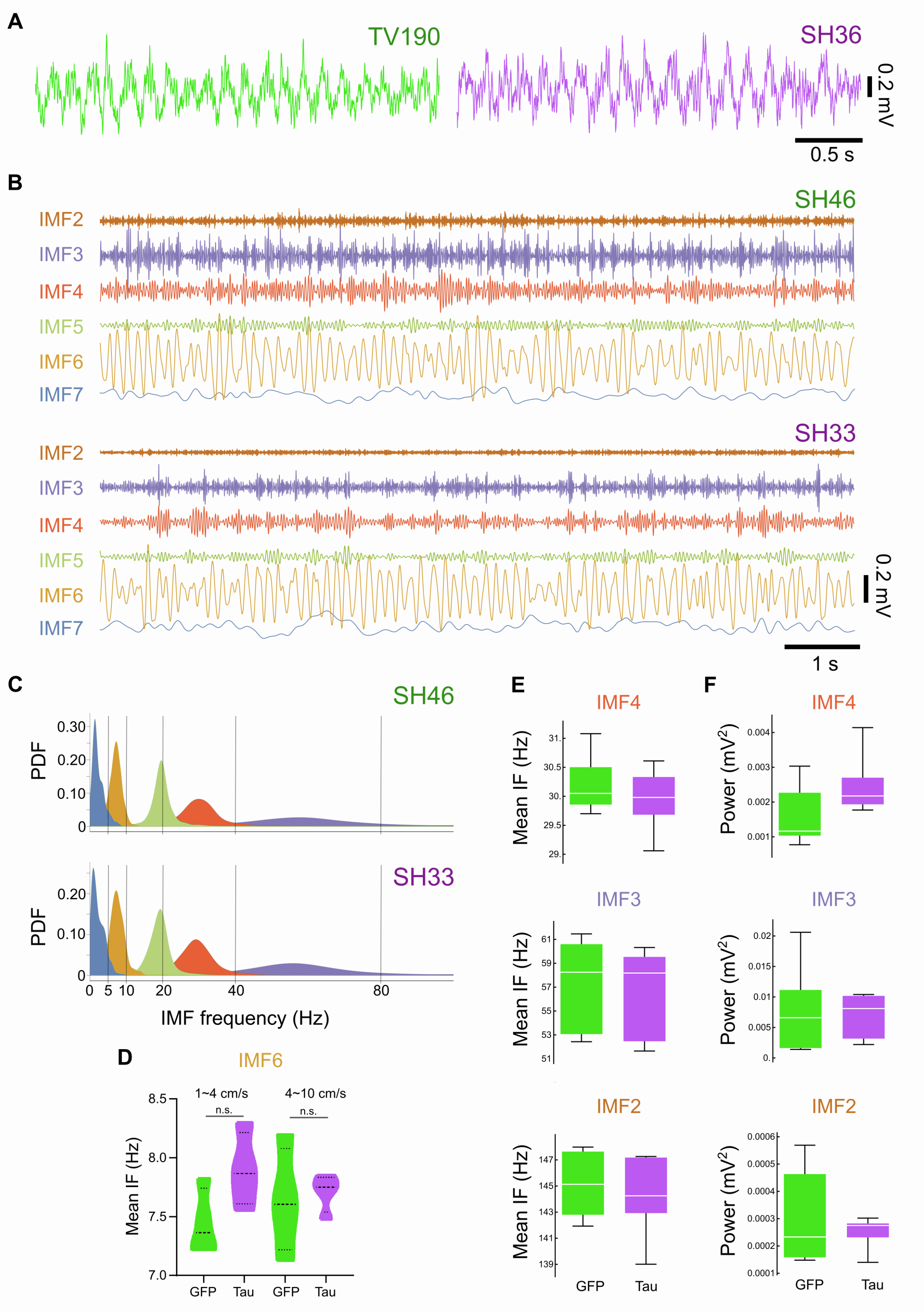
A
TV190
SH36
0.2 mV
0.5 s
B
SH46
IMF2
IMF3
IMF4
IMF5
IMF6
IMF7
SH33
IMF2
IMF3
IMF4
IMF5
IMF6
IMF7
0.2 mV
1 s
C
SH46
PDF
0.30
0.20
0.10
0
SH33
PDF
0.20
0.10
0
0 5 10 20 40 80
IMF frequency (Hz)
D
IMF6
1~4 cm/s
4~10 cm/s
n.s.
n.s.
Mean IF (Hz)
8.5
8.0
7.5
7.0
GFP Tau GFP Tau
E
IMF4
Mean IF (Hz)
IMF3
Mean IF (Hz)
IMF2
Mean IF (Hz)
GFP Tau
F
IMF4
Power (mV²)
IMF3
Power (mV²)
IMF2
Power (mV²)
GFP Tau

Figure S4. Unimpaired hippocampal theta and gamma oscillations in ADn-tau mice.

(**A**) LFPs recorded in the pyramidal cell layer of hippocampal CA1 during movement for an ADn-gfp mouse (TV190) and an ADn-tau mouse (SH36). (**B**) Intrinsic mode functions (IMFs, see Methods) obtained from CA1 LFPs. Top, ADn-gfp mouse (SH46). Bottom, ADn-tau mouse (SH33). Oscillations: IMF2, fast-gamma; IMF3, mid-gamma; IMF4, slow-gamma; IMF5, beta; IMF6, theta; IMF7, delta. (**C**) Histograms of instantaneous frequencies for IMF3 – IMF7 for the two cells from D. PDF, probability density function. (**D**) Distribution chart of mean instantaneous frequency (IF) values for 4 ADn-gfp mice (GFP, green) and 4 ADn-tau mice (Tau, purple) for two speed bins (1–4 cm/s, $t_{(6)}$ = 2.139, p = 0.0763; 4–10 cm/s, $t_{(6)}$ = 0.3, p = 0.7688) during spontaneous movement on the running disc (IMF6, theta); n.s, not significant. (**E**) Box-whisker plots of the mean IF for n=5 ADn-gfp mice (SH46, SH56, TV190, SH73, SH74) and n=5 ADn-tau mice (SH33, SH36, SH54, TV189, SH79). Mann-Whitney tests: IMF4, U = 15, p = 0.6761; IMF3, U = 15, p = 0.6761; IMF2, U = 15, p = 0.6761. (**F**) Mean power (from instantaneous amplitude) for the same cells as in E. IMF4, U = 6, p = 0.1437; IMF3, U = 9, p = 0.4034; IMF2, U = 13, p = 1. Data are represented as median and IQR (D-F).
